# Different Intermolecular Interactions Drive Nonpathogenic Liquid–Liquid Phase Separation and Potentially Pathogenic Fibril Formation by TDP-43

**DOI:** 10.3390/ijms232315227

**Published:** 2022-12-03

**Authors:** Yu-Teng Zeng, Lu-Lu Bi, Xiao-Feng Zhuo, Ling-Yun Yang, Bo Sun, Jun-Xia Lu

**Affiliations:** 1 School of Life Science and Technology, ShanghaiTech University, Shanghai 201210, China; 2 iHuman Institute, ShanghaiTech University, Shanghai 201210, China

**Keywords:** TDP-43, liquid–liquid phase separation, solution-state NMR

## Abstract

The liquid–liquid phase separation (LLPS) of proteins has been found ubiquitously in eukaryotic cells, and is critical in the control of many biological processes by forming a temporary condensed phase with different bimolecular components. TDP-43 is recruited to stress granules in cells and is the main component of TDP-43 granules and proteinaceous amyloid inclusions in patients with amyotrophic lateral sclerosis (ALS). TDP-43 low complexity domain (LCD) is able to de-mix in solution, forming the protein condensed droplets, and amyloid aggregates would form from the droplets after incubation. The molecular interactions regulating TDP-43 LCD LLPS were investigated at the protein fusion equilibrium stage, when the droplets stopped growing after incubation. We found the molecules in the droplet were still liquid-like, but with enhanced intermolecular helix–helix interactions. The protein would only start to aggregate after a lag time and aggregate slower than at the condition when the protein does not phase separately into the droplets, or the molecules have a reduced intermolecular helix–helix interaction. In the protein condensed droplets, a structural transition intermediate toward protein aggregation was discovered involving a decrease in the intermolecular helix–helix interaction and a reduction in the helicity. Our results therefore indicate that different intermolecular interactions drive LLPS and fibril formation. The discovery that TDP-43 LCD aggregation was faster through the pathway without the first protein phase separation supports that LLPS and the intermolecular helical interaction could help maintain the stability of TDP-43 LCD.

## 1. Introduction

Eukaryotic cells contain micro-scale compartments that are formed by specific proteins and nucleic acid through condensation. These compartments do not have bound membranes and are referred to as membraneless organelles, regulating diverse processes in cells [1,2]. Examples include stress granules formed when translation initiation is impaired in response to cellular stresses [3], P granules of C. elegans embryos [4], and Cajal bodies [5], etc. The dynamic equilibrium between the condensation and dissolution of these membraneless organelles are mainly controlled by the multivalent but weak interactions of constituting proteins and other biomolecules within the organelles [1]. Proteins with intrinsic disordered low complexity domain (LCD) are often the major components forming these membraneless organelles [6,7]. In vitro, these proteins with multivalency could de-mix from solution and form liquid droplets through a protein liquid–liquid phase separation (LLPS) process. Interestingly, many of these proteins can also form amyloid, which in most cases can be neurotoxic [8]. 

TDP-43 (transactive response (TAR) element DNA-binding protein of 43 kDa) is a nuclear ribonucleoprotein. It participates in many processes of RNA regulations and is able to autoregulate its own expression [9]. It is recruited to stress granules in cytoplasm, which contains other proteins with LCD such as GTPase activating protein1 (SH3 domain) (G3BP1), T cell intracellular antigen-1 (TIA-1), etc. [3]. Although TDP-43 is not an obligatory stress granule component, it modulates stress granule formation and disassembly [10]. The full-length TDP-43 protein contains 414 residues, with a nuclear localization signal (NLS) and two RNA-recognition motifs at the N-terminal region, but a low complexity domain (LCD) at the C-terminal region (267–414) [11]. The first RNA-recognition domain and the LCD are responsible for recruiting TDP-43 into stress granules [9]. In solution, the TDP-43 LCD domain [12,13] is able to form liquid droplets by phase separation. However, it can also aggregate into amyloids in common solutions and in cells [14]. TDP-43 aggregation is considered as the hallmark for amyotrophic lateral sclerosis (ALS) [10,15]. The protein inclusions are also sometimes found in patients with frontotemporal dementia (FTD) and other neurodegenerative diseases [9]. These diseases related to the formation of the pathological TDP-43 granules and the abnormal TDP-43 aggregation are considered as TDP-43 proteinopathies. To understand the mechanisms in regulating TDP-43, LLPS and aggregation would be essential for us to design molecules to delay TDP-43 aggregation, but not affect its LLPS. Most ALS-associated mutations of TDP-43 are located at LCD, indicating the importance of this domain [16]. The mechanisms determining TDP-43 LCD LLPS have been extensively investigated but questions remain. For example, LCDs involved in LLPS are usually intrinsically disordered and are composed of polar amino acids punctuated by aromatic residues (TDP-43 LCD sequence analysis in Table S1a–c) [17]. However, the TDP-43 LCD contains one secondary structure element of two short α-helices connected by a bend (residue 320–343) [14]. The TDP-43 LCD sequence contains a higher amount of hydrophobic residues (Table S1a) than most LCD proteins (LLPSDB-Statistics (bio-comp.org.cn accessed on 1 November 2022), with the helices mostly hydrophobic (Table S1c). The intermolecular helix–helix interactions driven by the hydrophobic interactions have been shown as the major forces for TDP-43 phase separation. however, this segment also belongs to the amyloidogenic core domain. The droplets TDP-43 LCD forms tend to be relatively small, about 1 μm in diameter in certain conditions (in our hand and also observed by other groups [6,13]), unlike in other phase separation proteins [6]. It is also not easy for us to find the condition to observe the on-going liquid droplet fusion events by differential interference contrast (DIC) microscopy. These indicate that the protein fusion equilibrium occurs quickly after the protein is dissolved in solution when the liquid droplet is very small. Because of these special properties, TDP-43 LCD is a good model to study for a better understanding of the interplay of different structural components in determining the protein condensation and aggregation.

Aside from the helical intermolecular interactions, other interactions have also been found to play roles in TDP-43 LCD LLPS. The two flanking regions on both sides of the helices are intrinsically disordered, referred as the intrinsic disordered region (IDR) (IDR1, (276-320) and IDR2, (343-414)). IDR2 contains a Gln/Asn-rich (QN) (344-360) motif immediately after the helices (320–343), with the sidechain amide bonds of Q, N residues able to participate in H-bonding, promoting the protein assembly or the amyloid formation [18,19,20]. Both IDR1 and IDR2 regions contain X-G/S, G/S-X sequence motifs (X represents the aromatic residues Phe, Trp, Tyr). The sidechain H-bonding is provided by the hydroxyl group of serine, the π–π stacking of aromatic rings or ionic charge–π interactions are all believed to contribute to the intermolecular interactions in protein phase separation [17,21,22]. For example, TDP-43 contains three Trp residues. All three Trp residues were found to play roles in regulating the protein LLPS by mutation studies, with the most important Trp located in the helical region [14]. TDP-43 LCD also contains several charged residues including five Args (Table S1) with a protein PI (isoelectric point) value of 10.78, and the pH of the solution controls its phase separation ability. A decrease in pH to 4–5 could diminish its phase separation completely while increasing the pH of solution to 6 and above would cause protein droplet formation [23,24]. Increasing the NaCl concentration also increases the protein LLPS by providing the electrostatic shielding effects [12,24]. Both the pH and salt effect indicate that the electrostatic repulsion inhibits its phase separation [25]. Therefore, there must exist an intricate balance between different interactions, which fine tunes the status of TDP-43 in solution. However, the relative contributions of these interactions to TDP-43 LCD LLPS are not well understood.

In this research, by screening different conditions, we investigated the special role of the helical region (320–343) and compared it to the rest of the sequence to understand how the balance of the contributions from these two parts of the sequence controlled the LLPS and what happened when the TDP-43 LCD stopped fusion. A structural transition intermediate toward aggregation was also discovered involving a decrease in the intermolecular helix–helix interaction and a reduction in the helicity. Using TDP-43 LCD as a model, our work provides a better understanding of the multivalence controlling the intermolecular interactions and protein phase separation.

## 2. Results

### 2.1. TDP-16 Phase Separation Is Very Sensitive to the Solution Conditions

#### 2.1.1. TDP-43 LCD Droplets Fusion Events Can Only Be Observed in a Narrow Concentration Range

**Figure 1:**
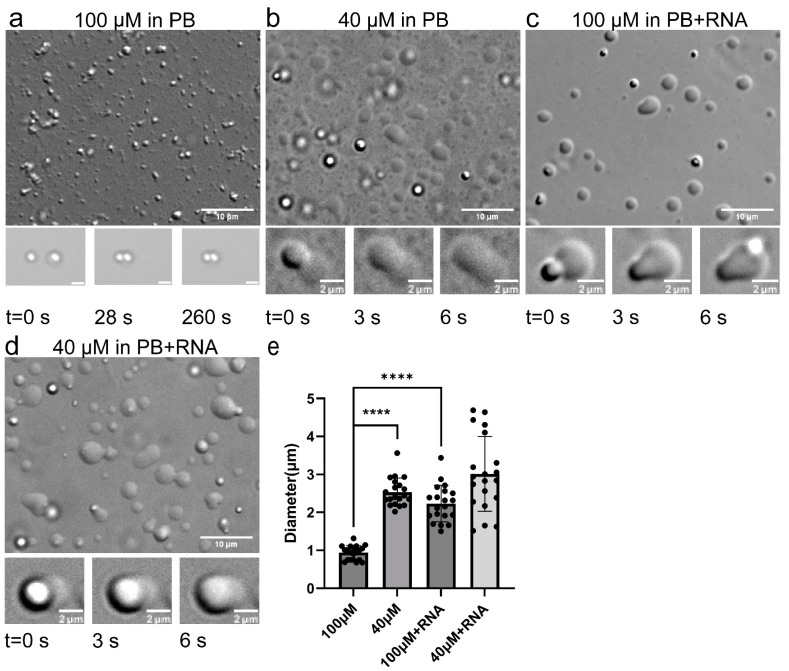
Images of TDP-43 LCD LLPS in four different solution conditions by DIC microscopy. (**a**) 100 μM in PB, 100 μM protein concentration in pH 6.0 10 mM phosphate buffer; (**b**) 40 μM in PB, 40 μM protein concentration in the same PB buffer; (**c**) 100 μM in PB + RNA, 100 μM protein concentration in pH 6.0, 10 mM phosphate buffer with yeast RNA (20 ng/μL); (**d**) 40 μM in PB + RNA, 40 μM protein concentration in the same PB buffer with RNA. Scale bars are 10 μm for the larger images. Representative events of TDP-43 LCD droplet fusion are shown at the bottom panel with the scale bar of 2 μm. The bottom panel of (**a**) shows snapshots from the optical tweezer experiment. (**e**) Statistical analysis of TDP-43 LCD droplet sizes for the four conditions. For each condition, 20 droplets were analyzed from a single image. Statistical differences were determined using an unpaired Student’s *t*-test; **** *p* < 0.0001 (GraphPad Prism).

The liquid–liquid phase separation (LLPS) of TDP-43 LCD (residue 267–414) was first investigated in 10 mM phosphate buffer (PB), pH 6.0. A 100 μM protein solution was prepared from dry protein powder and the droplets were less than 1 μm in diameter, as shown in Figure 1a. The turbidity of the solution was determined using an OD600 of about 0.47 (Figure S1a). A high resolution image was also taken using TEM, where the stained image showed many black spheres with a diameter of 1 μm or less (Figure S1b). Interestingly, we were not able to detect a very clear droplet fusion event (a short movie in Video S1), indicating that the active fusion has already finished before it could be observed. The droplets were able to stick to each other, but remained as separate particles. Controlled fusion was therefore carried out using optical tweezers to check the fusion ability of the droplets (snapshots shown in Figure 1a, bottom panel and the video shown in Video S2). In the video, two droplets were forced to touch each other for as long as 260 s (force profiles are shown in Figure S1c), but failed to fuse into one droplet. The results confirmed that the droplets formed at this condition had a low capability to fuse at this stage. We referred to these droplets as mature droplets.

Droplets formed at other protein concentrations were further tested. A higher concentration protein solution (200 μM, Figure S2a, Video S3a) did not show a clear droplet fusion either and a lower concentration protein solution (20 μM, Figure S2b, Video S4) only displayed some fusion events. Interestingly, the OD600 readings were low (~0.2) for both the 20 μM and 200 μM concentrations (Figure S2c), indicating that the solution turbidity was not positively correlated to the protein concentration. The turbidity was influenced by a combined effect of the particle number and size in the solution. At a 200 μM concentration, the solution looked much clearer than that at 100 μM, and became more turbid once diluted to a 100 μM concentration. A video showing the dilution process is displayed in Video S3b. It took 1–2 min for the buffer to diffuse and a change of equilibrium to take effect, showing more and bigger droplets forming upon dilution. The fusion event was best observed at a 40 μM concentration (Figure 1b, Video S5). The protein liquid droplets were also bigger at this condition, about 2–4 μm in diameter.

#### 2.1.2. RNA Enhanced TDP-43 LCD Droplet Fusion

The phase separation property was then investigated in the same PB condition with additional RNA (a final concentration of 20 c). Larger droplets were formed with the diameter ranging from 1 to 3 μm (Figure 1c). TEM also displayed many black spheres (Figure S1b). Figure 1c, lower panel and Video S6a show the fusion events. Video S6b shows the process of adding yeast RNA to the protein solution in real-time. It took 3 min for the RNA to show its effect and a change in equilibrium was displayed by the size of the droplet growing overtime. Therefore, adding RNA to the TDP-43 LCD solution would increase the size of the droplets and significantly alter the protein LLPS. The OD600 was about 0.51 (Figure S1a), a little larger than the reading without RNA. The fusion events were also observed for the 40 μM TDP-43 LCD solution in the presence of RNA (Figure 1d, Video S7). Larger droplets were also observed (1–5 μM in diameter).

### 2.2. The Intermolecular Interactions of TDP-43 LCD Monitored by Solution NMR

#### 2.2.1. TDP-43 LCD in Mature Droplets Is Still Mobile and Liquid-like

**Figure 2:**
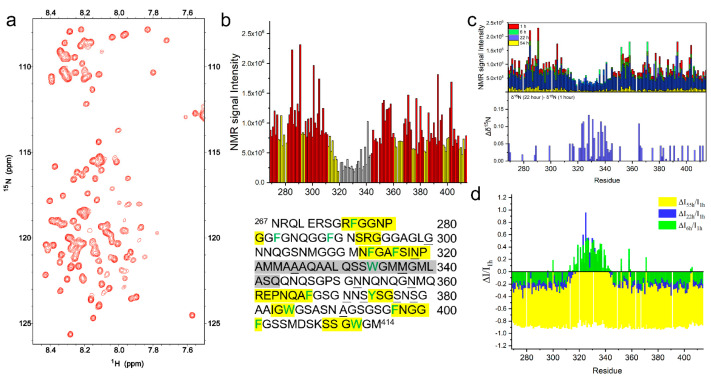
The ^1^H-^15^N HSQC spectra of TDP-43 LCD. (**a**) The ^1^H−^15^N HSQC spectra of TDP-43 LCD obtained within 2.5 h after the sample preparation at a concentration of 100 μM in pH 6.0, 10 mM phosphate buffer, and 25 °C. (**b**) The NMR peak intensity for each residue obtained from the ^1^H−^15^N HSQC shown in (**a**). Helical region of the TDP-43 LCD (in gray) exhibited the lowest signal intensity, while several short segments (in yellow) also exhibited mild signal loss. The amino acid sequence of TDP-43 LCD was shown at the bottom panel with the corresponding highlighted sequences in gray and in yellow. The aromatic amino acids were in green, and the underlined residues were those showing a higher intensity in the pH 5.5 MES buffer. (**c**) (Top) Comparison of the NMR signal intensity from ^1^H−^15^N HSQC spectra of TDP-43 LCD (100 μM) in pH 6.0, 10 mM phosphate buffer, obtained within 2.5 h from the sample preparation (labeled as I_1h_ using the approximate starting time, in red) and obtained at ~6 h (I_6h_, in green), 22 h (I_22h_, in blue), or 53 h (I_55h_, in yellow) from the sample preparation. (Bottom) ^15^N chemical shift differences (Δδ^15^N) during TDP−16 fibrillation in PB. Δδ^15^N = δ^15^N (22nd hour spectrum in PB) −δ^15^N (1^st^ hour spectrum in PB). (**d**) NMR signal loss ratio (ΔI/I_1h_) over time, where ΔI represents the NMR signal intensity after ~6 h, 22 h, or 53 h (I_6h_, I_22h_ or I_55h_) minus I_1h_.

The droplets formed by TDP-43 LCD had very little ability to fuse at the concentration of 100 μM in PB quickly after dissolution. The ^1^H-^15^N HSQC spectrum of the freshly prepared TDP-43 LCD (100 μM) in PB was therefore obtained to investigate the molecular status of this condition. The protein will slowly aggregate under this solution condition. The protein aggregation was monitored using THT fluorescence. Figure S3a indicates that the THT fluorescence remained at a low level for about 200 min before it increased. The THT tended to bind to the protein aggregates with amyloid properties, giving out fluorescence. Therefore, the result suggests a lag time of at least 200 min before the protein started to aggregate. The spectrum was taken and finished within the lagging period (Figure 2a). The spectra chemical shift assignment was based on the reported work with BMRB accession code: 26823, which was carried out on 20 μM TDP-43 (residue 267–414) in pH 6.1, 10% D_2_O 20 mM, MES buffer [12]. Table S2 compares four of the reported chemical shifts including BMRB 26823, indicating that the chemical shifts were slightly different depending on the sample conditions. Our results matched with BMRB 26823 the best. Table S3 includes all of the chemical shifts from ^1^H-^15^N HSQC obtained for different conditions in our work.

The peak intensity of TDP-43 LCD in PB buffer is shown in Figure 2b, displaying a significant signal decrease in the helical region at residue 320–343 (in gray) compared to the rest of the sequence. This is consistent with previous research reports that the helix is involved in intermolecular interactions, and the dynamic equilibrium causes the decrease of the NMR signal intensity in this region [12,21]. Aside from the helical region, we also observed a signal intensity decrease at several places in IDR1 and IDR2 labeled in yellow in Figure 2b, indicating those regions were also involved in the intermolecular interactions. These regions were mostly co-localized with the aromatic residues with X-G/S, G/S-X (X represented the aromatic residues Phe, Trp, Tyr, shown in green in the sequence). The observation was also reported by Pantoja-Uceda et al. on TDP-43 LCD at pH 4.0 [22]. The regions with decreased intensity were generally matched with their reports, but differences existed, since the solution condition was very different. The intermolecular interactions provided by these regions could probably be simplified using the so-called “stickers-and-spacers” model [17]. The regions were also overlapped with some peptides, named as LARKS (low-complexity aromatic-rich kinked segments) by Eisenberg et al. [26]. However, more complex interaction modes or fuzzy interactions will be needed to include all of the properties encoded in the IDR sequence [27,28].

**Figure 3:**
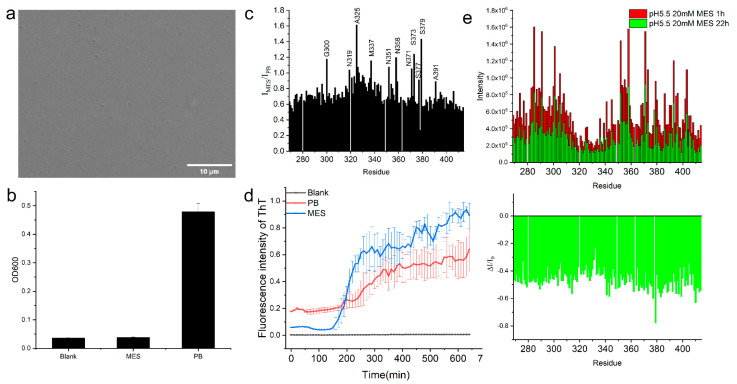
Experiment results on 70 μM TDP-43 LCD in pH 5.5 MES buffer, the LLPS condition. (**a**) DIC image of TDP-43 LCD in MES buffer. (**b**) OD600 value of 70 μM TDP-43 LCD in pH 5.5 MES buffer (MES) compared to the blank MES buffer and 100 μM TDP-43 LCD in pH 6.0 PB buffer (PB). (**c**) Comparison of the NMR signal intensities of ^1^H-^15^N HSQC of TDP-43 LCD between the LLPS+ condition (PB) and the LLPS condition (MES). (**d**) THT fluorescence changes of TDP-43 LCD in two different conditions (PB vs. MES). (**e**) (Top) Comparison of the NMR signal intensity from ^1^H-^15^N HSQC spectra of TDP-43 LCD in the LLP condition in MES (labeled as I_1h_ using the time approximate in the middle of the experiment, in red) and obtained at ~22 h (I_22h_, in green) from the sample preparation. (Bottom) NMR signal loss ratio (ΔI/I_1h_) over time, where ΔI represents the NMR signal intensity after 22 h minus I_1h_.

It was reported that MES buffer in pH 5.5 disfavored the phase separation [23], therefore, the protein molecules were supposed to be mostly in a low level of aggregation or liquid-like state. A total of 70 μM of TDP-43 LCD in pH 5.5, 20 mM MES buffer was prepared. A DIC microscopic image is displayed in Figure 3a (a video is shown in Video S8), showing very few protein droplets. An OD600 reading was close to the blank solution (Figure 3b). Our observation confirmed a low level of protein LLPS in the MES buffer. In order to investigate whether the molecules in the mature droplet in PB were in a liquid-like state, the ^1^H-^15^N HSQC spectrum of the TDP-43 LCD (70 μM) in pH 5.5 MES buffer was obtained for comparison. The chemical shift assignment was again based on the report with BMRB accession code: 26823, which was on a 20 μM protein MES buffer with a higher pH (6.1). The ^15^N chemical shift difference between these two conditions was minor, within ±0.05 ppm (Figure S3b). Figure S3c shows the peak intensities for TDP-43 LCD in MES, displaying similar intensity variations at those regions shown for the PB buffer condition. The signal intensity ratio between the two conditions is displayed in Figure 3c. The residue peak intensity in the MES buffer was around 0.6–0.7 of that in the PB buffer for most residues, except a few showing a higher intensity for MES. The intensity level was consistent with a lower concentration of protein in MES (70 μM in MES vs. 100 μM in PB buffer) for the NMR studies, indicating no aggregation of protein in PB. The residue at 320–343 representing the helical region of TDP-43 LCD showed a consistent higher intensity ratio than the other region in Figure 3c, which supported the conclusion that the helical intermolecular interactions were reduced in MES. Other higher intensity peaks in MES were labeled in Figure 3c and underlined in the sequence in Figure 2b, which scattered throughout the sequence. The high intensity indicated the molecular interactions involving these residues were significantly decreased in MES. The ^15^N chemical shift changes are shown in Figure S3d. A short region at the N-terminus and residue 320–343 displayed larger negative shifts (up to −0.15 ppm) when the buffer was changed from MES to PB. A negative change in the amide ^15^N chemical shift suggested an increased helical structure for the helical region in the PB condition [12]. Both the amide peak intensity and ^15^N chemical shift changed, and therefore, supports that the helix structure at 320–343 was involved in stronger intermolecular interactions in PB. However, the protein was still liquid-like without much decrease in the NMR peak intensity for most of the residues in the mature droplets.

#### 2.2.2. The Helical Intermolecular Interaction Was Reduced during TDP-43 LCD Fibrillation in PB

TDP-43 LCD started aggregation after about 200 min in PB (Figure S3a). The aggregation process was monitored using ^1^H-^15^N HSQC to see the changes at the residue level. Figure 2c (top) shows the changes in the spectra intensity with time at 100 μM protein concentration in the PB buffer compared to the spectra collected at the first hour (1st spectrum, Figure 2a), sixth hour, 22nd hour, and 53rd hour, respectively. The panel in Figure 2d displays the peak intensity ratio, comparing all three spectra to the first one, and showed a consistent decrease in the intensity for most of the residues during time. However, different changes were again observed around the helical region (residue 312–343, with the additional segment 312–319). At the sixth hour and 22nd hour, the peak intensity at residue 312–343 increased instead of decreasing. This indicates that the dynamic exchange caused by the helical intermolecular interactions were decreased at the beginning stages of the protein aggregation. The rest of the sequence (IDR1, IDR2), however, had a consistent decrease in intensity of about 20–30%, supporting the protein in slow aggregation. Afterward, at the 53rd hour, the last spectrum showed a significant decrease of about 90% for all residues. The ^15^N chemical shift changes were compared between the 22nd hour and the first hour spectra, shown in Figure 2c (bottom) with bigger positive changes (up to 0.13 ppm) mainly at the helical region. This supports a decrease in helicity during aggregation. Therefore, there would be a decrease in the intermolecular interaction mediated by the helices in the pathway for TDP-43 LCD fibrillation. The fibrillation initiated at the IDR1 and IDR2 regions since the signal of these regions decreased first.

The protein aggregation with 70 μM concentration in pH 5.5 MES was also studied, showing faster aggregation with only about 150 min of lag time indicated by the THT fluorescence (Figure 3d). The changes in the ^1^H-^15^N HSQC spectra intensity were also faster. At 22 h, the intensity for all residues decreased by about 50% (Figure 3e). Since the helical intermolecular interaction was weaker in this condition, the faster aggregation supports our conclusion that a decrease in the intermolecular interaction mediated by the helices facilitated the protein fibril aggregation. LLPS could be a protective state of the protein and reduce the protein aggregation to some degree.

### 2.3. TDP-43 LCD Aggregation Was Enhanced by an Introduction of 150 mM Urea to the Solution

**Figure 4:**
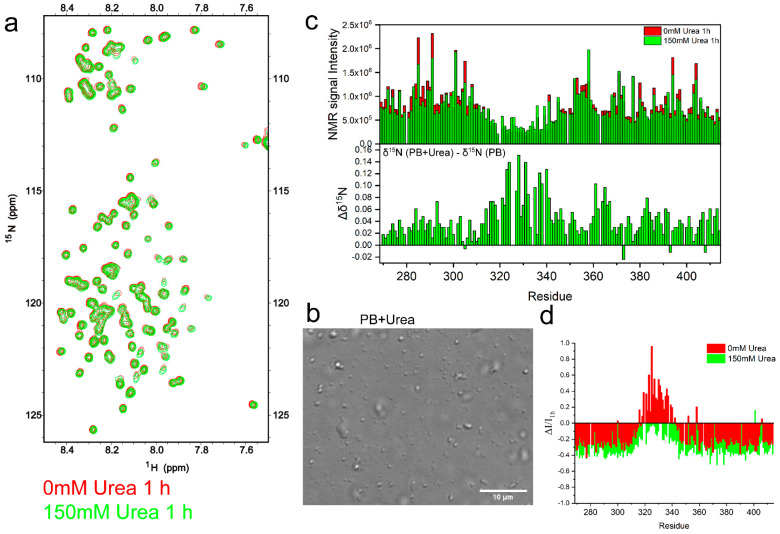
The effect of 150 mM urea on TDP-43 LCD liquid–liquid phase separation and protein aggregation. (**a**) The ^1^H-^15^N HSQC spectra of TDP-43 LCD obtained within 2 h from sample preparation at a protein concentration of 100 μM in pH 6.0, 10 mM phosphate buffer with 150 mM urea (in green) or without urea (in red). The temperature was 25 ℃. (**b**) DIC image of 100 μM TDP-43 LCD in PB buffer with 150 mM urea. (**c**) (Top) Comparison of the NMR signal intensity from ^1^H-^15^N HSQC spectra in (**a**) with (green) or without (red) urea. (Bottom) ^15^N chemical shift changes (Δδ^15^N) of TDP-43 LCD (100 μM) upon the addition of 150 mM urea in pH 6.0, 10 mM phosphate buffer. Δδ^15^N = δ^15^N (PB + Urea) −δ^15^N (PB). (**d**) NMR signal loss ratio (ΔI/I_1h_) over time. ΔI = I_22h_ (NMR signal intensity after ~22 h) −I_1h_. Samples contained 150 mM urea shown in green or without urea shown in red.

In order to further perturb the intermolecular interactions of protein molecules, 150 mM of urea was added to the TDP-43 LCD solution in PB. The ^1^H-^15^N HSQC experiment was carried out to investigate how urea changed the protein at the molecular level (Figure 4a). This sample showed smaller and less homogeneous spheres observed by DIC microscopy (Figure 4b). The OD600 reading was about 0.34 (Figure S1a), lower than that in PB. We did not observe very clear droplet fusion events (Video S9). Urea is a protein denaturing agent, which would probably change the protein structures and decrease the intermolecular helix–helix interactions of TDP-43 LCD, reducing the protein fusion ability. However, the protein displayed much faster fibril growth, as shown in Figure S3a, with about a 2-hour lag time (120 min).

The ^1^H-^15^N HSQC spectra intensities are summarized in Figure 4c (top), comparing the two conditions with or without urea. It showed a slight decrease in the intensity for non-helical regions upon adding 150 mM urea. However, it also displayed a slight increase in the intensity for the helical region, supporting the assumption that the helical intermolecular interaction was perturbed in the presence of urea. The changes in the ^15^N chemical shift are shown in Figure 4c (bottom), indicating positive shifts (up to 0.15 ppm) around the helical region, which suggested a decrease in helicity. Therefore, urea decreased the protein intermolecular interactions and disturbed the helical structure of TDP-43 LCD. Both effects would negatively impact the protein LLPS.

^T^he ^1^H-^15^N HSQC spectrum at the 22nd hour was also collected and the intensity is shown in Figure S3e. It shows that the peak intensity in the helical region decreased slightly, but the rest of the sequence displayed bigger decreases. The ratios of the intensity changes are shown in Figure 4d and compared to that in the PB buffer without urea. It showed a bigger decrease in the intensity by about 40% with urea and about 30% without urea. This is consistent with the faster fibrillation of TDP-43 with urea, as shown in Figure S3a. During aggregation of up to 22 h, the helical region showed a small decrease in the intensity in the presence of urea, but an increase in the intensity without urea. As discussed above, the fibrillation would involve an intermediate step of a partial release of the intermolecular interaction mediated by TDP-43 LCD helices, and a loosening of the helix structure. Both were already introduced upon the addition of urea at the time the sample was prepared. Therefore, only small changes in the intensity at the helix region were observed after 22 h in the presence of urea. The fibrillation was enhanced since the solution already went through this intermediate step upon the addition of urea.

### 2.4. The Disordered Region Showed a Low LLPS Ability and a Slightly Slower Aggregation Rate

#### 2.4.1. TDP-43 LCD Fragments without the Helices Have a Low LLPS Ability

**Figure 5:**
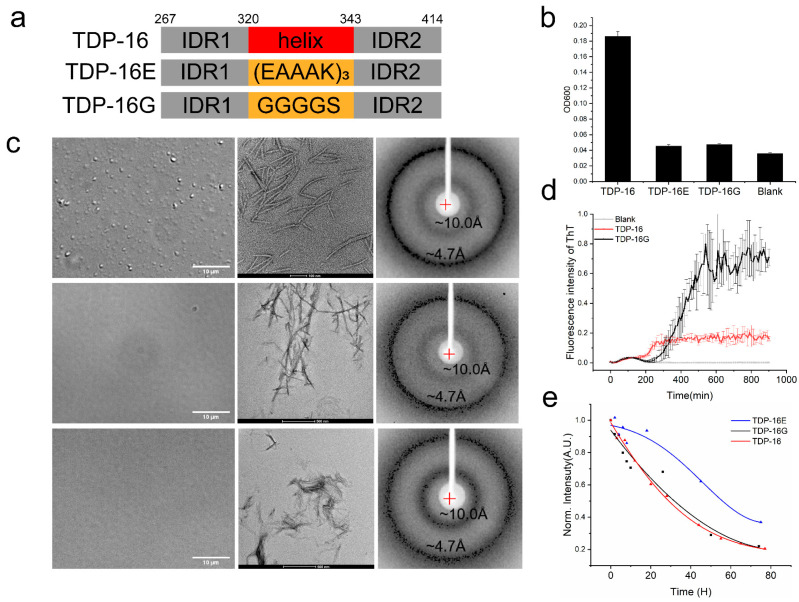
The function of the helical region of TDP-43 LCD in liquid–liquid phase separation and fibrillation. The protein was at a concentration of 20 μM in pH 6.0, 10 mM phosphate buffer for all samples. (**a**) Schematic representation of three domains of TDP-43 LCD (also known as TDP-16) and its mutants TDP-16E and TDP-16G. The helical domain was replaced by (EAAAK)_3_, a rigid helical sequence; for TDP-16E, the helical domain was replaced by GGGGS, a flexible disordered linker, for TDP-16G. (**b**) Turbidity (OD600 values) of the freshly prepared wild-type TDP-43 LCD and its mutants in PB buffer. Error bars represent the SD of three replicates. (**c**) Liquid–liquid phase separation of freshly prepared TDP-16, TDP-16E, and TDP-16G was imaged by DIC microscopy. The amyloid fibrils that formed were imaged by negative-staining TEM and identified by XRD. The fibrils were collected for study after 4 days of incubation of the protein solution. (**d**) The growth rate of the TDP-16 and TDP-16G fibrils monitored by THT fluorescence. Error bars represent the SD of three replicates. (**e**) Aggregation rate of TDP-16, TDP-16E, and TDP-16G monitored by the protein’s intrinsic fluorescence.

TDP-43 LCD is intrinsically disordered except for the short helical region. The α-helical segment mediates the intermolecular interactions and promotes the phase separation. However, our work also showed that the rest of the sequence, the IDR, contributed to the intermolecular interactions. In order to compare the contributions of the two parts in determining the protein phase separation properties, we replaced the α-helical segment with two sequences (Figure 5a). One was (EAAAK)_3_, which was as a designed helical structure, but did not promote the intermolecular helical–helical interaction [29] (the protein was labeled as TDP-16E). The other one was (GGGGS), which was designed to be a flexible linker to connect the two flanking IDR domains (the protein was labeled as TDP-16G and the wild-type TDP-43 LCD was labeled as TDP-16 in Figure 5). The protein solutions were prepared in the PB buffer in the 20 μM concentration. As shown in Figure 5b, the turbidity of the wild-type protein solution (OD600 = 0.18) was significantly greater than that of the mutant solutions (OD600 = 0.04). DIC images (Figure 5c, left panel) also confirmed the turbidity reading that only the wild-type solution displayed the protein droplets. The results confirm that the sequence of the α-helical segment has a dominant role in determining the phase separation of TDP-43 LCD, much stronger than that of the two IDRs.

#### 2.4.2. TDP-43 LCD Fragments without the Helices Still Form Fibril Aggregates

Although TDP-16E and TDP-16G have a low LLPS ability, a long incubation at room temperature would also lead to protein aggregation. The TEM images of the protein aggregates after 4 days of incubation are shown in Figure 5c, middle panel, which also displayed fibril-like images. The x-Ray diffraction of the collected aggregates is shown in Figure 5c, right panel. All three displayed two diffraction rings at about 4.7 Å and 10.0 Å, supporting the formation of amyloid fibrils. THT fluorescence was applied to monitor the aggregation process (Figure 5d). The TDP-43 LCD had a lag time of about 200 min before the fluorescence started to increase. TDP-16G had a lag time of about 220 min, then a quick increase in THT fluorescence. The absolute intensity of THT fluorescence was not compared since different fibrils would emit fluorescence differently. TDP-16E aggregates were not sensitive to THT binding without a significant fluorescence reading. Therefore, the intrinsic fluorescence of the protein was also monitored to observe the aggregation of TDP-43 LCD and its mutants (Figure 5e). During the protein aggregation, the intrinsic fluorescence was decreased. The experiments indicated a faster intensity decrease in wild-type TDP-43 LCD and TDP-16G than TDP-16E (with a rigid helix (EAAAK)_3_). However, the wild-type protein (with the amyloidogenic helices) and TDP-16G (without the helices) had a similar rate of intensity decrease. The result was slightly different from the THT binding experimental result, since the two experiments observed different aspects of protein aggregation (fibril binding THT enhances fluorescence signals and the protein aggregation decreases its intrinsic fluorescence signal.) The results indicate that IDR regions contributed significantly to the aggregation while the amyloidogenic helices had a small effect, modulating the aggregation rate.

### 2.5. RNA Enhanced TDP-43 LCD LLPS Mainly by Mediating the Intermolecular Interactions with IDR1 and IDR2

**Figure 6:**
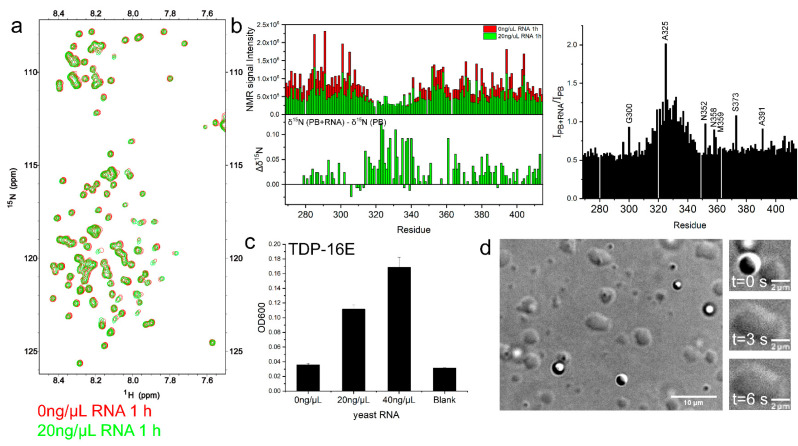
Effect of yeast RNA on the TDP-43 LCD liquid–liquid phase separation and protein aggregation. (**a**) The ^1^H-^15^N HSQC spectra of 100 μM TDP-43 LCD in pH 6.0, 10 mM phosphate buffer with 20 ng/μL yeast RNA (in green) or without yeast RNA (in red), obtained within 2 h from sample preparation. The temperature was 25 °C. (**b**) (Left) The NMR signal intensity comparison of the ^1^H-^15^N HSQC of TDP-43 LCD (100 μM) in pH 6.0, 10 mM phosphate buffer with (in green) or without 20 ng/μL yeast RNA (in red) and ^15^N chemical shift changes of TDP-16 (100 μM) upon adding 20 ng/μL yeast RNA in pH 6.0, 10 mM phosphate buffer. Δδ^15^N = δ^15^N (PB + RNA) −δ^15^N (PB). (Right) The NMR signal intensity ratio shown for all residues. (**c**) Turbidity (OD600 values) of the mutant TDP-16E (100 μM) with different concentrations of yeast RNA. Error bars represent SD of three replicates. (**d**) Liquid-liquid phase separation of TDP-16E (100 μM) with a concentration of 20 ng/μL yeast RNA detected by DIC microscopy. The right panel of (**d**) shows liquid-like droplet fusion in three time steps.

In order to understand how RNA interacted with TDP-43 LCD and alters the LLPS, we obtained the ^1^H-^15^N HSQC spectrum of TDP-43 LCD (100 μM) in the presence of 20 ng/μL RNA (Figure 6a) and compared to the protein spectrum in PB buffer. The protein aggregation in the presence of RNA showed a similar lag time (~200 min) as the PB buffer condition (Figure S3a), and the spectrum was taken within the lag time. The signal intensity is displayed in Figure 6b for all of the residues. It showed a consistent signal decrease for all of the residues, except for the helical region, clearly indicating that RNA mostly affected the disordered region. The intensity ratio between the with and without RNA was higher, close to 1.0 around the helical region, indicating the helical region was less affected by RNA binding (Figure 6b, right). The changes in the ^15^N chemical shift to the positive direction (up to 0.12 ppm) centered around the helical region indicate a decrease in the helicity (Figure 6b, bottom).

In order to further demonstrate the RNA effect on protein LLPS, different amounts of yeast RNA (0–40 ng/μL) were added to TDP-16E (100 μM). This showed a consistent increase in OD600 when the RNA concentration was increased (Figure 6c). DIC microscopy also displayed more and larger protein droplets and clear droplet fusion events (Figure 6d, Video S10). Without RNA, TDP-16E showed little LLPS in the PB buffer and the disordered regions IDR1 and IDR2 had a very weak power in inducing LLPS compared to the helical region. However, the addition of RNA enhanced the interactions with the IDR, which compensated for the lack of the intermolecular interactions of the rigid helices.

### 2.6. The Dynamic Changes of Protein Molecules Probed by ^19^F NMR

**Figure 7:**
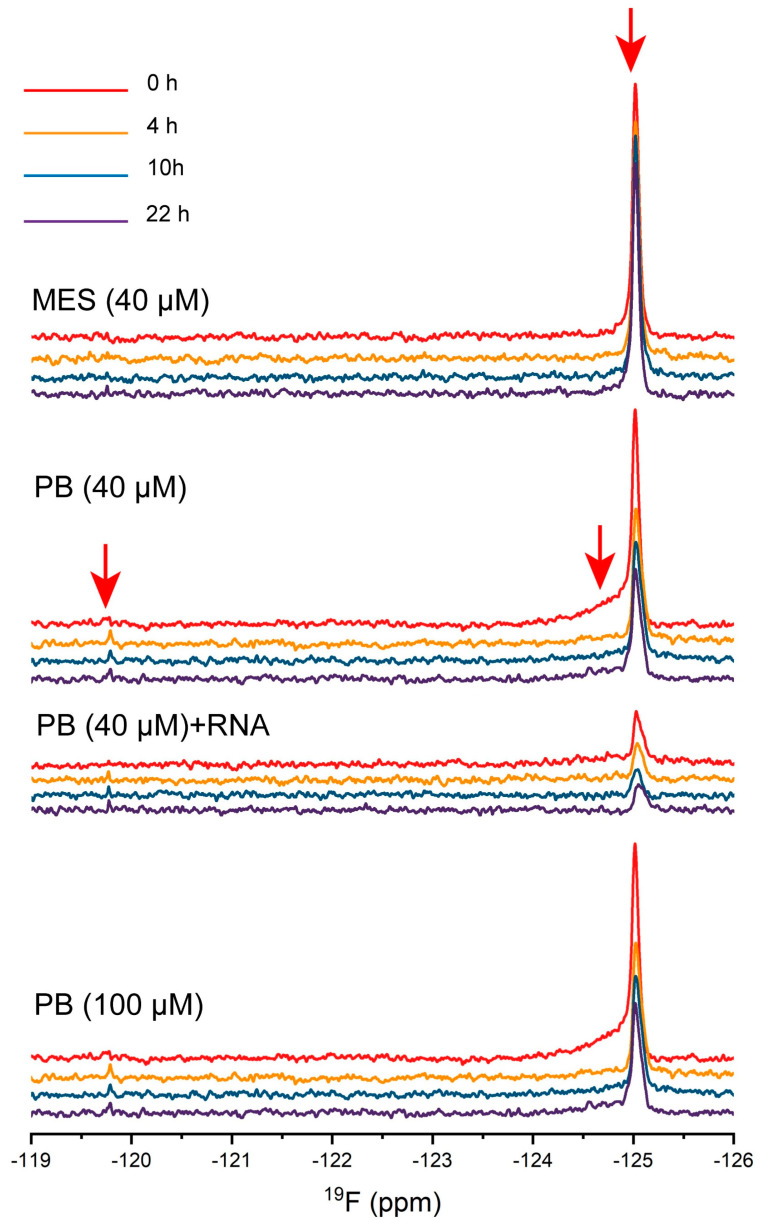
The dynamic changes of the protein molecules probed by ^19^F NMR. Time course spectra of 40 μM protein in pH 5.5, 20 mM MES buffer, 40 μM protein in pH 6.0, 10 mM PB buffer, 40 μM protein with 20 ng/μL in pH 6.0, 10 mM PB buffer, and 100 μM protein in pH 6.0, 10 mM PB buffer are shown. Spectra obtained at ~30 min (labeled as 0 h, in red), 4 h (in orange), 10 h (in blue), and 22 h (in purple) from the sample preparation. The temperature was 25 °C.

^19^F NMR has been used to study protein aggregation for its high sensitivity. It allows for the detection of aggregation intermediates for Aβ [30] and other amyloids [31]. The TDP-43 LCD has three Trp residues, with one located at the helical region (Trp334) and two at IDR2 (Trp385, Trp412). The three Trp residues play roles in modulating protein LLPS, and the Trp334Gly mutation reduced the protein LLPS most significantly [14]. The three Trp residues were replaced by 5-fluoro-tryotophan, thus a change in the sidechains of the three Trp residues was observed. Four conditions were compared including 40 μM protein in pH 5.5, 20 mM MES buffer, 40 μM protein in pH 6.0, 10 mM PB buffer, 40 μM protein with 20 ng/μL RNA in pH 6.0, 10 mM PB buffer, and 100 μM protein in pH 6.0 10, mM PB buffer (Figure 7). The freshly prepared sample showed only one dominant ^19^F peak at −125.0 ppm for all conditions, but clearly with a difference. The protein in MES showed the narrowest (<0.1 ppm linewidth at the half height) and highest peak, while the addition of RNA to the solution significantly reduced the intensity of the peak. Both 40 μM and 100 μM protein solutions in PB showed similar spectra, with a similar intensity and a broad shoulder peak at −124.75 ppm. The signal intensity in PB was slightly weaker than that in MES. The results confirmed that in the MES buffer, the three Trp residues were very dynamic and had a similar chemical environment, supporting a low LLPS in the MES buffer. The molecular dynamics were reduced for the PB buffer condition, especially for the 100 μM protein concentration, causing the weaker signals. The PB buffer condition was the LLPS active condition, and the shoulder peak for the 40 μM protein concentration was mostly from a contribution of Trp334 at the helical region since the helical interaction was the most dominant effect in inducing LLPS. The remaining sharp peak at −125.0 ppm was not attenuated significantly for the 40 μM condition, but was attenuated significantly for the 100 μM protein concentration considering a 2.5 times protein concentration. The observation also indicated stronger intermolecular interactions involving the helices and IDR. Three Trp residues were the least dynamic in the presence of RNA and support a stronger interaction between RNA and IDR in promoting LLPS. Although a clear difference was observed here on the freshly prepared samples (40 μM protein concentration) in PB with or without RNA, the DIC images showed active LLPS for both.

The ^19^F NMR spectra also changed as a function of time. The peak intensity at −125.0 ppm was attenuated clearly for the two PB buffer conditions without RNA. An additional peak at −119.8 ppm slowly appeared, but remained very weak for all three PB solutions. This peak was likely a peak for the protein oligomer intermediate during the aggregation, but was not investigated here.

## 3. Discussion

### 3.1. A Fine Tune of the Different Interactions Affects the Protein LLPS Equilibrium and Droplet Sizes

In this research, we showed ways to manipulate LLPS and the liquid droplet sizes of TDP-43 LCD. By reducing the protein concentrations or adding RNA, the LLPS equilibrium was disturbed. The sizes of the droplet could be increased and the fusion events could be observed by DIC only in a narrow protein concentration range. The active fusion event actually indicates a non-equilibrium situation, where more protein molecules are recruited to the droplet. Protein LLPS requires multivalence and a balance between different intermolecular interactions. To simplify the situation, the interactions can be tentatively put into two groups here for TDP-43 LCD: one is from the helices and the other is from the rest sequence, the IDR sequences. The two groups of interactions could induce the molecules into a type of loosely associated network, connecting the molecules in the helical region and the IDR regions. At 100 μM protein concentration in the PB buffer condition, the intermolecular interaction mediated by the helices was probably too strong, but the interactions mediated by the IDR were too weak, not enough to extend the molecular network, therefore, the LLPS stopped at a very early stage with small droplet sizes. Decreasing the protein concentration to 40 μM could shift the dynamic equilibrium and reduce the helix–helix intermolecular interactions so that the different intermolecular interactions could be more compatible with each other in intensity, and the protein droplet sizes increase. Similarly, adding RNA enhances the interactions mediated by IDR sequences to make them more compatible in the intensity to the helical interactions, and the protein droplet sizes increase.

The real stress granules or TDP-43 granules in cells contain a greater variety of molecules including RNA and full-length TDP-43. In this real situation, the TDP-43 LCD helix–helix interaction may not be so dominant if the protein concentration is lower. Some other proteins found in stress granules such as G3BP1, hnRNPA2, etc. also contain LCD and would interact with TDP-43 and RNA through a similar interaction mechanism, maintaining the stability. Therefore, the principle gained here should still be applicable in a more complicated system, although more components would have to be taken into consideration.

### 3.2. The Molecular Status of Proteins in the Mature Droplets

The molecular properties of the mature droplets were also investigated. Since the mature droplets did not fuse in pH 6.0 PB buffer at the 100 μM protein concentration, the molecules may not still be liquid-like. The ^1^H-^15^N HSQC spectra intensity of freshly prepared mature droplets was first compared to the MES condition at 70 μM protein concentration, showing that the signal intensity was proportional to the protein concentration for most residues at IDR (Figure 3c). Protein was soluble, and showed very low LLPS in the MES condition. Therefore, the protein molecules in the mature droplets were still liquid-like, with similar dynamic properties as the ones in MES. Using THT fluorescence, we found that the protein aggregation lag time was 200 min for a 100 μM protein concentration in PB (Figure S3a), indicating that the protein was not severely aggregated within this time frame. Although the mature droplet did not fuse, changes in the solution condition would make the situation change quickly such as dilution into a buffer or adding RNA. The observations could also indicate that the molecules in the mature droplet were still in an active equilibrium, and not in a severe aggregation state. A mature protein droplet should have a certain lifetime before the protein aggregation, considering its function in the cell. The membraneless organelle was supposed to have an active function and able to dissolve upon regulation.

Although the ^1^H-^15^N HSQC spectra showed that the molecules still maintained high dynamic motions, in general, in the mature droplet, the intermolecular helical interaction was stronger, displayed by a lower intensity of the peak and negative ^15^N chemical shift changes in the PB buffer. We also found that the ^19^F signal intensity from the Trp aromatic ring was attenuated (in PB vs. in MES) and not proportional to the protein concentrations (40 μM vs. 100 μM in PB), indicating that the aromatic residue sidechains were involved in the intermolecular interactions for LLPS. Therefore, the protein molecules in the mature droplet were involved in stronger intermolecular interactions, but were still liquid-like and able to change its molecular interactions quickly upon induction.

### 3.3. TDP-43 LCD Aggregation Intermediate

When the protein aggregates, it usually accompanies a NMR signal loss for all of the residues, which was observed for the 100 μM protein concentration in PB after 53 h (Figure 2c). It was unexpected for us to observe a signal increase in the helical region during the protein aggregation while the signal decrease in the IDR region was clearly seen (spectra taken at the sixth hour and 22nd hour, Figure 2c). The signal increase suggested an enhancement in the molecular dynamics of the helical region, probably through a slight release of the helix–helix interaction. Therefore, we observed an intermediate step during TDP-43 LCD aggregation from the protein droplets. We then found two other conditions with reduced helix–helix interaction that indeed showed faster aggregation with shorter lag time studied using THT fluorescence (70 μM protein concentration in pH 5.5 MES and 100 μM protein concentration, 150 mM urea in pH 6.0 PB). The ^1^H-^15^N HSQC spectra also showed a similar observation that the two conditions with less helical intermolecular interactions displayed a faster decay in the signal intensity. We observed that TDP-16G (without the amyloidogenic helices) aggregated at a similar rate as or at a slightly slower rate than the wild-type (with the helices), suggesting that IDR regions contributed significantly to the aggregation. Therefore, our observation supports that the protein fibrillation started at the IDR regions, which was followed by the structural conversion of the helical region. The amyloidogenic helical region has high multiplicity of binding modes [27,28], which would contribute to the stability of the aggregates after the structural transition by forming ordered amyloid structures.

Previous NMR studies have indicated that for residue 321-330, the α-helical structure only populated about 50% of the conformational ensemble, while for residue 331-343, the helical population was even smaller [12]. Although the values were obtained mostly at a 20 μM protein concentration at pH 6.1 MES, where the protein was not in droplets, it indeed showed that the helices in TDP-43 LCD were in dynamic exchange in the conformation, and not a strong helical structure. Previous research also indicated increased helical structure upon protein LLPS, consistent with our results [32]. Therefore, a proper helix–helix interaction is needed for protein LLPS and at the same time, the intermolecular interaction also helps maintain the stability of the helical structure. Without good intermolecular interaction, the helical structure can easily become loose and the protein is easy to aggregate, as shown in the urea condition or our pH 5.5 MES condition. However, even in the mature droplet state, for a long incubation time, there are still opportunities for a temporary breakup of the helix–helix interaction and a slight loosening of the helicity. This would explain the increase in the HSQC spectra intensity at the helical region during the protein aggregation after a long incubation time. This observation was consistent with studies on the cell, showing that the recruitment of TDP-43 into granules would protect the protein from fast aggregation [32,33]. Some of the ALS-associated mutations were also reported to alter the helix–helix interactions or the helical propensity [34]. Our observation again reinforces the importance of the helices for TDP-43 LCD LLPS and aggregation.

### 3.4. The Polar, Aromatic Residue Rich Sequence of Low Complexity Domain

X-G/S and G/S-X (X represents the aromatic residues) sequences have been found in many proteins with LLPS. Interestingly, the nucleoporins in nuclear pore complexes also contain many phenylalanyl-glycyl (FG)-rich repeats at their selective filter for the random fuzzy interactions with their cargo, the transport factors [35]. Similar observations on the NMR signal intensity attenuation were also reported on this FG-rich region of nucleoporins. TDP-43 was originally expressed in the nucleus and was transported to the cytoplasm through the nuclear pore complexes. We speculate that the intermolecular interactions between TDP-43 LCD and the nucleoporins may also be present during the transportation of TDP-43 out of the nucleus.

In conclusion, this research studied the protein molecular properties when TDP-43 LCD formed mature droplets. They were still liquid-like, although the intermolecular interactions were stronger than the lower protein concentration conditions or no LLPS conditions. The protein exit and reentrant equilibrium could be shifted by modifying the solution environment, here, the addition of adding RNA or dilution of the protein was demonstrated. The protein in the mature droplets would aggregate gradually, but the aggregation was slower than some conditions with decreased helix–helix intermolecular interactions. A partial loosening of the helical intermolecular interaction was identified as the aggregation intermediate step. Not all interactions were probed here, and the studies were only carried out using a very simplified system to exclude other influence factors. Recently, fuzzy interactions and the multiplicity-of-binding modes have been recognized as the framework to explain and predict the propensity of the proteins to form droplets or amyloids based on the sequence (https://fuzdrop.bio.unipd.it, accessed on 1 November 2022). IDR sequences of TDP-43 LCD sample mostly disordered interactions favoring the protein droplet formation. The information gained could provide useful guidance to design ligands to fine tune the protein phase behaviors.

## 4. Materials and Methods

### 4.1. Expression and Purification

The cDNA of human TDP-43 LCD (from residue 267 to 414) was derived from plasmid encoded thioredoxin (Trx)-fused TDP-43 LCD (gift from Prof. Hong-Yu Hu), the cDNA of mutant TDP-16E and TDP-16G were synthesized directly, and all cDNA were cloned into Pet32M with the N-terminal hexa-His-tag. Proteins were overexpressed in E. coli BL21(DE3) or Rosetta (DE3). The uniformly labeled peptide was expressed in M9 minimal medium containing 4 g of glucose and 1.5 g of ^15^NH_4_Cl per liter. Unlabeled peptide was expressed in the LB medium. Cells were grown at 37 °C until the OD600 reading was 0.8. Then, the protein expression was induced overnight at 22 °C by adding 0.5 mM isopropyl β-D-thiogalactoside (IPTG) for TDP-43 LCD, or induced 6 h at 37 °C by adding 1 mM IPTG for TDP-16E and TDP-16G.

The 5-fluoro-tryptpphan (5FW) labeled peptide was obtained by adding 5-fluoroindole (5FI) in M9 minimal medium. In brief, after cells were grown to OD600 of about 0.6 in 1 L M9 minimal medium, cells were centrifuged and the pellets were transferred into 0.5 L M9 minimal medium containing 2 g of glucose, 0.75 g of NH_4_Cl, 30 mg of 5FI, 20 mg of Tyr, 20 mg of Phe, and 0.5 g glyphosate (to suppress the pentose phosphate pathway for aromatic amino acid synthesis). Following incubation for 30 min, 0.5 mM (IPTG) was added to initiate protein expression.

Cells were collected by centrifugation at 10,000× *g* for 10 min and the cell pellet was resuspended in 100 mL lysis buffer (50 mM Tris-HCl, 300 mM NaCl, pH 8.0) with 1 mM PMSF, lysed by French press, and centrifuged at 28,000× *g* for 30 min, then the peptides in the inclusion bodies were washed with water, and resuspended in 20 mL denaturing binding buffer (50 mM Tris-HCl, 300 mM NaCl, 8 M urea, pH 8.0) until most inclusion bodies dissolved at 4 °C. Further centrifugation at 28,000× *g* for 10 min, 4 °C, and the supernatant was purified by the Ni-NTA affinity column, while the elution buffer contained 8 M urea and 500 mM imidazole.

For TDP-43 LCD, the protein solution with 8 M urea and 500 mM imidazole was dialyzed in water for 1 day at room temperature. All dialysates were collected and lyophilized. The dried sample was then dissolved in 30% formic acid and subsequently purified by reverse-phase HPLC on a C18 column eluted by a water–acetonitrile solvent system. The HPLC elution containing pure recombinant proteins was lyophilized and stored at −80 °C for further experiments.

For TDP-16E and TDP-16E, the protein was initially stored in 8 M urea and desalted into the phosphate buffer with a 0.5 mL ZebaSpin Desalting column (Thermo Scientific, Carlsbad, CA, USA) and diluted to 100 uM or 20 uM for the experiments.

### 4.2. Turbidity Measurements

TDP-43 LCD and its variants were dissolved in different buffers at 25 °C and incubated for 5 min. A total of 100 μL of samples were transferred to a 96-cell plate. Turbidity was measured using a plate reader (Enspire, PerkinElmer, Waltham, MA, USA) monitoring the absorbance at 600 nm. The tested solution conditions included the PB buffer, pH 6.0, 10 mM phosphate buffer; PB + RNA, pH 6.0, 10 mM phosphate buffer with yeast RNA (20 ng/uL, Sigma, St. Louis, MO, USA); PB + urea, pH 6.0, 10 mM phosphate buffer with 150 mM urea; pH 5.5, 20 mM MES buffer.

### 4.3. Thioflavin-T Assays

TDP-43 LCD and its variants were dissolved in different buffers containing 20 μM THT at 25 °C and transferred to a 96-cell plate. The fluorescence emission at 480 nm was measured using a plate reader (Enspire, PerkinElmer, USA) with an excitation wavelength at 430 nm [14]. Five seconds of shaking was applied before each reading. The blank was pH 6.0, 10 mM phosphate buffer only.

### 4.4. Intrinsic Fluorescence Spectroscopy

A total of 20 μM of TDP-43 LCD and its variants were dissolved in a phosphate buffer and 150 μL of the samples were transferred to a cuvette (Quartz SUPRASIL, Hellma, Mannheim, Germany). For the fluorescence spectroscopy measurements (FluoroMax-4, HORIBA, Edison, NJ, USA), the excitation wavelength was set to 295 nm, and the emission wavelength range was 310–500 nm. Both slits were 5 nm, and the scanning step was 1 nm [36,37]. The variation in the fluorescence maximum intensity with time indicates the aggregation rate.

### 4.5. Differential Interference Contrast (DIC) Microscopy

TDP-43 LCD and its variants were dissolved in different buffers at 25 °C and incubated for 5 min. For all samples, 5 μL of protein solutions were dropped onto the bottom of a glass dish. Then, the solution was checked by an inverted microscope (Nikon ECLIPSE Ti, Nikon, Tokyo, Japan) and imaged by a digital camera (ORCA-Flash 4.0, HAMAMATSU, Hamamatsu, Japan) with a 60 × 1.49 NA oil objective. The blank was pH 6.0, 10 mM phosphate buffer only.

### 4.6. Optical Tweezers

An optical tweezer microscope C-trap^TM^ from LUMICKS (Amsterdam, The Netherlands) with two steerable traps was used to perform the controlled fusion of droplets [38]. A total of 100 μM of TDP-43 LCD was dissolved in a phosphate buffer and droplets were formed in minutes. These droplets flowed into the chamber just before data acquisition. A 1064 nm laser with a low light intensity (<0.5 W) was applied to minimize heating. One droplet was held in place by a trap, and the other steerable trap was used to capture other droplets and bring them toward the stationary droplet with a velocity of 0.04 μM s^−1^ until the surface of the two droplets touched [39]. Force-extension and image data were taken at 5 Hz. Touch times were determined via analysis from the laser signal and confirmed with video signals.

### 4.7. Negative-Staining Transmission Electron Microscope (TEM)

To observe the droplets, 100 μM of TDP-43 LCD was dissolved in different buffers and incubated for 5 min. To observe the fibril, 20 μM of TDP-43 LCD and its variants were incubated for 4 days before imaging. In total, 5 µL of the sample solution was adsorbed to the glow-discharged TEM grid (Cu, 300 mesh; Beijing Zhongjingkeyi Technology Co., Ltd., Beijing, China) for 45 s. Then, the grid was washed using 5 μL of water for 3 s, and finally stained with 5 μL of 2% uranyl acetate for 45 s. The TEM images were obtained using a transmission electron microscope (Talos L120C, FEI, Brno, Czech). The acceleration voltage was 120 KeV. The exposure time for each image was 2 s.

### 4.8. X-ray Diffraction (XRD)

A total of 20 μM of TDP-43 LCD and its variants were incubated first for 4 days. The solution mixtures were then centrifuged at 50,000 rpm for 2 h (Optima Max-TL, BECKMAN COULTER, Bera, CA, USA) and the pellets were collected. The precipitation was applied to a Single Crystal X-ray Diffraction instrument (Bruker D8 VENTURE, Bruker, Karlsruhe, Germany, Germany) for the measurement and the light source was Cu Kα radiation at a 1.54184Å wavelength [40].

### 4.9. Solution-State NMR

The samples were dissolved in 90% H_2_0/10% D_2_O pH 6.0 phosphate buffer with or without RNA/urea or pH 5.5 MES buffer. All 2D ^1^H-^15^N HSQC NMR experiments were recorded on a Bruker 800 M Hz AVANCE III spectrometer at 298 K. The spectrum was first taken within 2 h from the sample preparation and more spectra were taken again after ~6, 22, or 53 h. All spectra were collected with the following parameters: 128* and 2048* complex pairs in the indirect ^15^N and direct ^1^H dimensions, 32 scans, 13.9 and 28 ppm as the spectral widths for ^1^H and ^15^N, respectively. Each experimental time was approximately 1 h 52 min. The ^1^H-^15^N HSQC peak assignments were based on a published chemical shift list deposited in BMRB under the accession code 26823. A summary of the ^1^H and ^15^N chemical shifts from four different publications are listed in Table S2 to show the similarity and difference between the different samples (BMRB code: 26823, 50154, 26728, 26816).

Proton chemical shifts were directly referenced using DSS on a TDP-43 LCD sample prepared for this purpose, and the ^15^N chemical shifts were referenced indirectly. All spectra were processed using either Sparky or Topspin 4.1.3. All chemical shifts gained from the ^1^H-^15^N HSQC spectra with various conditions prepared in this work are reported in Table S3.

### 4.10. ^19^F NMR Spectroscopy

The samples were dissolved in 90% H_2_0/10% D_2_O pH 6.0 phosphate buffer with or without RNA or pH 5.5 MES buffer. All NMR spectra were recorded on a Bruker AVANCE-600 MHz spectrometer (Bruker Biospin, Billerica, MA, USA) at 298 K. The spectrum was first taken within ~30 min from the sample preparation and more spectra were taken again after ~4, 10, or 22 h. All spectra were collected with the following parameters: 40960 complex points and 1024 scans. Each experimental time was approximately 30 min. All samples contained TFA as an internal reference, which was set at −75.6 ppm. Line broadening of 10 Hz was used to process the final spectra. Origin 2018 and MestReNova were used to plot the data.

## Data Availability

The data presented in this study are available in supplementary material.

## Supplementary Materials

The following supporting information can be downloaded at: https://www.mdpi.com/article/10.3390/ijms232315227/s1.

## References

[1] Banani S.F., Lee H.O., Hyman A.A., Rosen M.K. (2017). Biomolecular condensates: Organizers of cellular biochemistry. Nat. Rev. Mol. Cell Biol..

[2] Wang B., Zhang L., Dai T., Qin Z., Lu H., Zhang L., Zhou F. (2021). Liquid-liquid phase separation in human health and diseases. Signal Transduct. Target. Ther..

[3] Buchan J.R., Parker R. (2009). Eukaryotic stress granules: The ins and outs of translation. Mol. Cell.

[4] Brangwynne C.P., Eckmann C.R., Courson D.S., Rybarska A., Hoege C., Gharakhani J., Jülicher F., Hyman A.A. (2009). Germline P granules are liquid droplets that localize by controlled dissolution/condensation. Science.

[5] Machyna M., Heyn P., Neugebauer K.M. (2013). Cajal bodies: Where form meets function. Wiley Interdiscip. Rev. RNA.

[6] Molliex A., Temirov J., Lee J., Coughlin M., Kanagaraj A.P., Kim H.J., Mittag T., Taylor J.P. (2015). Phase separation by low complexity domains promotes stress granule assembly and drives pathological fibrillization. Cell.

[7] Antifeeva I.A., Fonin A.V., Fefilova A.S., Stepanenko O.V., Povarova O.I., Silonov S.A., Kuznetsova I.M., Uversky V.N., Turoverov K.K. (2022). Liquid-liquid phase separation as an organizing principle of intracellular space: Overview of the evolution of the cell compartmentalization concept. Cell. Mol. Life Sci..

[8] Vendruscolo M., Fuxreiter M. (2022). Protein condensation diseases: Therapeutic opportunities. Nat. Commun..

[9] Tziortzouda P., Van Den Bosch L., Hirth F. (2021). Triad of TDP43 control in neurodegeneration: Autoregulation, localization and aggregation. Nat. Rev. Neurosci..

[10] Dewey C.M., Cenik B., Sephton C.F., Johnson B.A., Herz J., Yu G. (2012). TDP-43 aggregation in neurodegeneration: Are stress granules the key?. Brain Res..

[11] François-Moutal L., Perez-Miller S., Scott D.D., Miranda V.G., Mollasalehi N., Khanna M. (2019). Structural Insights Into TDP-43 and Effects of Post-translational Modifications. Front. Mol. Neurosci..

[12] Conicella A.E., Zerze G.H., Mittal J., Fawzi N.L. (2016). ALS Mutations Disrupt Phase Separation Mediated by alpha-Helical Structure in the TDP-43 Low-Complexity C-Terminal Domain. Structure.

[13] Li H.R., Chen T.C., Hsiao C.L., Shi L., Chou C.Y., Huang J.R. (2017). The physical forces mediating self-association and phase-separation in the C-terminal domain of TDP-43. Biochim. Biophys. Acta.

[14] Jiang L.L., Che M.X., Zhao J., Zhou C.J., Xie M.Y., Li H.Y., He J.H., Hu H.Y. (2013). Structural transformation of the amyloidogenic core region of TDP-43 protein initiates its aggregation and cytoplasmic inclusion. J. Biol. Chem..

[15] Sun Y., Chakrabartty A. (2017). Phase to Phase with TDP-43. Biochemistry.

[16] Buratti E. (2015). Functional Significance of TDP-43 Mutations in Disease. Adv. Genet..

[17] Martin E.W., Holehouse A.S., Peran I., Farag M., Incicco J.J., Bremer A., Grace C.R., Soranno A., Pappu R.V., Mittag T. (2020). Valence and patterning of aromatic residues determine the phase behavior of prion-like domains. Science.

[18] Reijns M.A., Alexander R.D., Spiller M.P., Beggs J.D. (2008). A role for Q/N-rich aggregation-prone regions in P-body localization. J. Cell Sci..

[19] Fan H.C., Ho L.I., Chi C.S., Chen S.J., Peng G.S., Chan T.M., Lin S.Z., Harn H.J. (2014). Polyglutamine (PolyQ) diseases: Genetics to treatments. Cell Transplant..

[20] Mompeán M., Hervás R., Xu Y., Tran T.H., Guarnaccia C., Buratti E., Baralle F., Tong L., Carrión-Vázquez M., McDermott A.E. (2015). Structural Evidence of Amyloid Fibril Formation in the Putative Aggregation Domain of TDP-43. J. Phys. Chem. Lett..

[21] Murray D.T., Tycko R. (2020). Side Chain Hydrogen-Bonding Interactions within Amyloid-like Fibrils Formed by the Low-Complexity Domain of FUS: Evidence from Solid State Nuclear Magnetic Resonance Spectroscopy. Biochemistry.

[22] Pantoja-Uceda D., Stuani C., Laurents D.V., McDermott A.E., Buratti E., Mompeán M. (2021). Phe-Gly motifs drive fibrillization of TDP-43’s prion-like domain condensates. PLoS Biol..

[23] Pakravan D., Michiels E., Bratek-Skicki A., De Decker M., Van Lindt J., Alsteens D., Derclaye S., Van Damme P., Schymkowitz J., Rousseau F. (2021). Liquid-Liquid Phase Separation Enhances TDP-43 LCD Aggregation but Delays Seeded Aggregation. Biomolecules.

[24] Lim L., Wei Y., Lu Y., Song J. (2016). ALS-Causing Mutations Significantly Perturb the Self-Assembly and Interaction with Nucleic Acid of the Intrinsically Disordered Prion-Like Domain of TDP-43. PLoS Biol..

[25] Mompeán M., Chakrabartty A., Buratti E., Laurents D.V. (2016). Electrostatic Repulsion Governs TDP-43 C-terminal Domain Aggregation. PLoS Biol..

[26] Guenther E.L., Cao Q., Trinh H., Lu J., Sawaya M.R., Cascio D., Boyer D.R., Rodriguez J.A., Hughes M.P., Eisenberg D.S. (2018). Atomic structures of TDP-43 LCD segments and insights into reversible or pathogenic aggregation. Nat. Struct. Mol. Biol..

[27] Gianni S., Freiberger M.I., Jemth P., Ferreiro D.U., Wolynes P.G., Fuxreiter M. (2021). Fuzziness and Frustration in the Energy Landscape of Protein Folding, Function, and Assembly. Acc. Chem. Res..

[28] Vendruscolo M., Fuxreiter M. (2022). Sequence Determinants of the Aggregation of Proteins Within Condensates Generated by Liquid-liquid Phase Separation. J. Mol. Biol..

[29] Chen X., Zaro J.L., Shen W.C. (2013). Fusion protein linkers: Property, design and functionality. Adv. Drug Deliv. Rev..

[30] Suzuki Y., Brender J.R., Soper M.T., Krishnamoorthy J., Zhou Y., Ruotolo B.T., Kotov N.A., Ramamoorthy A., Marsh E.N.G. (2013). Resolution of oligomeric species during the aggregation of Abeta1-40 using (19)F NMR. Biochemistry.

[31] Larda S.T., Simonetti K., Al-Abdul-Wahid M.S., Sharpe S., Prosser R.S. (2013). Dynamic equilibria between monomeric and oligomeric misfolded states of the mammalian prion protein measured by 19F NMR. J. Am. Chem. Soc..

[32] Gasset-Rosa F., Lu S., Yu H., Chen C., Melamed Z.E., Guo L., Shorter J., Da Cruz S., Cleveland D.W. (2019). Cytoplasmic TDP-43 De-mixing Independent of Stress Granules Drives Inhibition of Nuclear Import, Loss of Nuclear TDP-43, and Cell Death. Neuron.

[33] Mann J.R., Gleixner A.M., Mauna J.C., Gomes E., DeChellis-Marks M.R., Needham P.G., Copley K.E., Hurtle B., Portz B., Pyles N.J. (2019). RNA Binding Antagonizes Neurotoxic Phase Transitions of TDP-43. Neuron.

[34] Conicella A.E., Dignon G.L., Zerze G.H., Schmidt H.B., D’Ordine A.M., Kim Y.C., Rohatgi R., Ayala Y.M., Mittal J., Fawzi N.L. (2020). TDP-43 alpha-helical structure tunes liquid-liquid phase separation and function. Proc. Natl. Acad. Sci. USA.

[35] Hough L.E., Dutta K., Sparks S., Temel D.B., Kamal A., Tetenbaum-Novatt J., Rout M.P., Cowburn D. (2015). The molecular mechanism of nuclear transport revealed by atomic-scale measurements. Elife.

[36] Zhuo X.F., Wang J., Zhang J., Jiang L.L., Hu H.Y., Lu J.X. (2020). Solid-State NMR Reveals the Structural Transformation of the TDP-43 Amyloidogenic Region upon Fibrillation. J. Am. Chem. Soc..

[37] Ladokhin A.S. (2000). Fluorescence Spectroscopy in Peptide and Protein Analysis. Encyclopedia of Analytical Chemistry: Applications, Theory and Instrumentation.

[38] Patel A., Lee H.O., Jawerth L., Maharana S., Jahnel M., Hein M.Y., Stoynov S., Mahamid J., Saha S., Franzmann T.M. (2015). A Liquid-to-Solid Phase Transition of the ALS Protein FUS Accelerated by Disease Mutation. Cell.

[39] Gui X., Luo F., Li Y., Zhou H., Qin Z., Liu Z., Gu J., Xie M., Zhao K., Dai B. (2019). Structural basis for reversible amyloids of hnRNPA1 elucidates their role in stress granule assembly. Nat. Commun..

[40] Zhang J., Wang J., Ma C., Lu J. (2020). Hydroxyapatite Formation Coexists with Amyloid-like Self-Assembly of Human Amelogenin. Int. J. Mol. Sci..

