# Supplementary material for: Different Intermolecular Interactions Drive Nonpathogenic Liquid–Liquid Phase Separation and Potentially Pathogenic Fibril Formation by TDP-43
Source: Int J Mol Sci. 2022 Dec 3;23(23):15227. doi: 10.3390/ijms232315227 (PMC9741235; doi:10.3390/ijms232315227)
Supplement: Supplementary file 1 [file ijms-23-15227-s001.zip › ijms_supplementinformation-edited.pdf]

Supporting Information for

Different intermolecular interactions drive nonpathogenic liquid-liquid phase separation and potentially pathogenic fibril formation by TDP-43

Yu-teng Zeng[1], Lu-lu Bi[1], Xiao-feng Zhuo[1], Ling-yun Yang[2], Bo Sun[1] and Jun-xia Lu[1]*
[1] School of Life Science and Technology, ShanghaiTech University, Shanghai, 201210, China
[2] iHuman Institute, ShanghaiTech University, Shanghai, 201210, China

*Jun-xia Lu.
Email: lujx@shanghaitech.edu.cn

This PDF file includes:

Figures S1 to S4
Tables S1 to S3
Legends for Movies S1 to S10

Other supporting materials for this manuscript include the following:

Movies S1 to S10
Chemical shifts and peak intensity table in .csv format corresponding to table S3
OD600 reading in .csv format corresponding to figure 3b, 5b, 6c and figure S1a, S2c.
The NMR resonance intensity ratio table in .csv format corresponding to figure 2c, figure 2d, figure 3e, figure S3e, figure 4d.

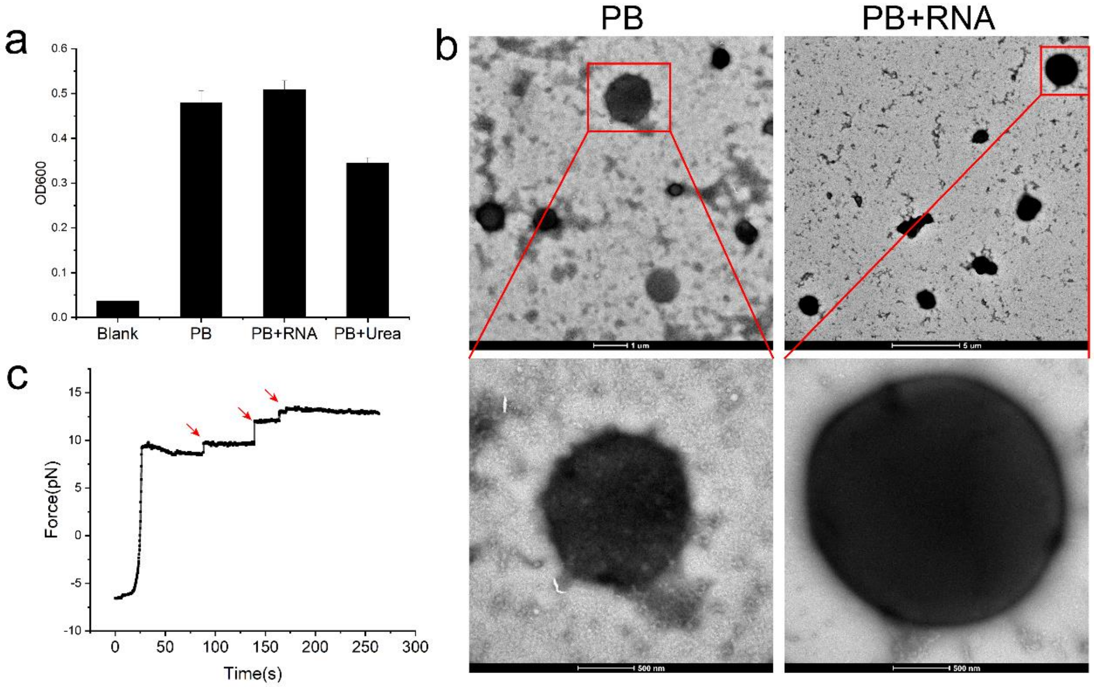

Figure S1

The comparison of TDP-43 LCD at different conditions. (a) Turbidity (OD600 values) of TDP-43 LCD solutions in four different conditions. (blank, pH 6.0 10 mM phosphate buffer; PB, 100 μM protein concentration in pH 6.0 10 mM phosphate buffer; PB+RNA, 100 μM protein concentration in pH 6.0 10 mM phosphate buffer with yeast RNA (20ng/μL); PB+Urea, 100 μM protein concentration in the same PB buffer and 150mM urea). Error bars represent SD of three replicates. **(b)** Liquid-like droplets were imaged by negative-staining TEM, while enlarged images were presented below. (PB, pH 6.0 10mM phosphate buffer; PB+RNA, pH 6.0 10mM phosphate buffer with yeast RNA (20ng/μL). Both samples were at a concentration of 100 μM. **(c)** Force profile of the right droplet shown in figure 1a bottom panel during the forced fusion process was recorded in VideoS2 using the optical tweezer. Red arrows represented the many attempts forcing the droplets closer.

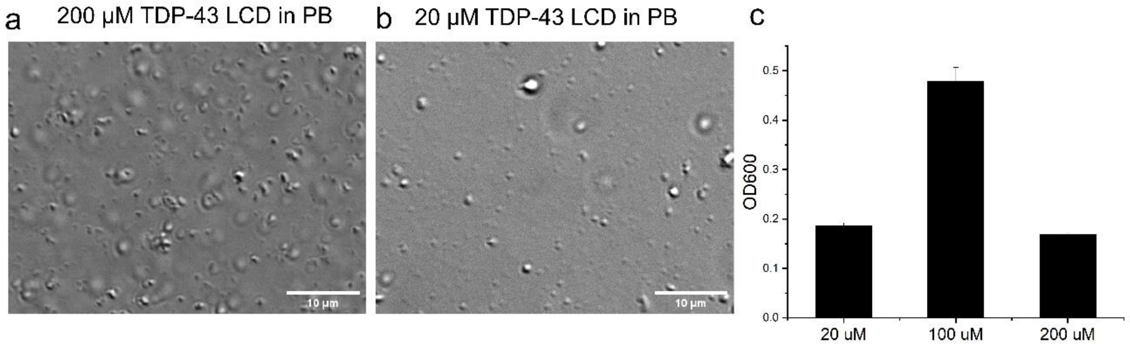

Figure S2

The concentration effect on TDP-43 LCD in PB buffer (a) Image of TDP-43 LCD LLPS in 200 μM protein concentration in pH 6.0 10 mM phosphate buffer (PB) by DIC microscopy; **(b)** Image of TDP-43 LCD LLPS in 200 μM protein concentration in pH 6.0 10 mM phosphate buffer (PB) by DIC microscopy; **(c)** Turbidity (OD600 values) of TDP-43 LCD solutions in three different protein concentrations in the same PB buffer. Error bars represent SD of three replicates.

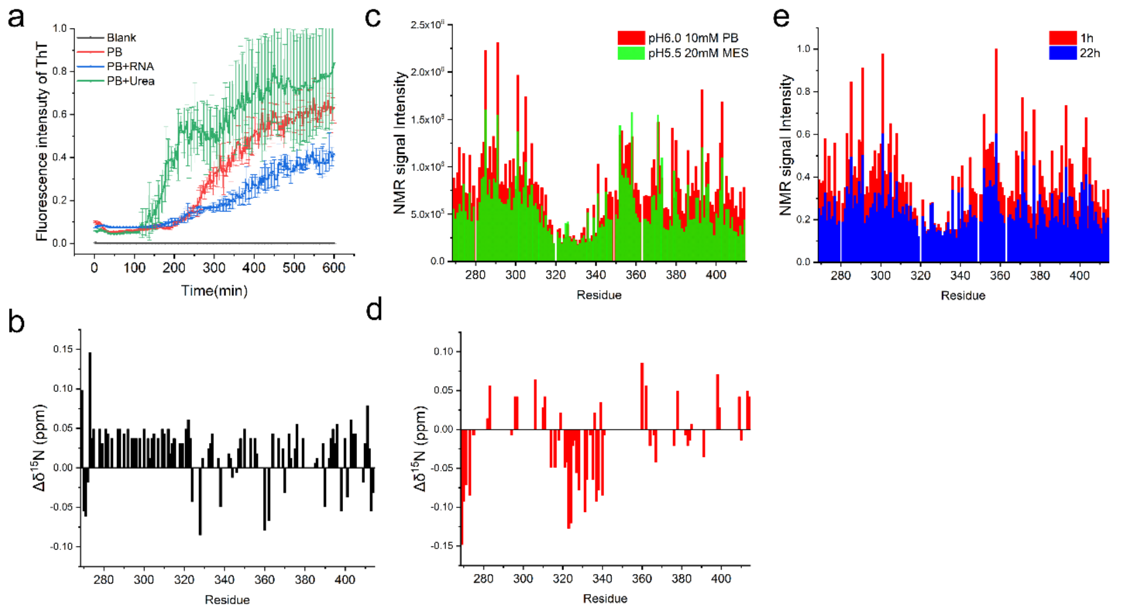

Figure S3

TDP-43 LCD ThT fluorescence changes in different conditions and its ^{1}H-^{15}N HSQC spectra comparison (a) ThT fluorescence changes of 100 μM TDP-43 LCD in different conditions. All solutions were in pH 6.0 10 mM phosphate buffer, with PB+RNA containing yeast RNA (20ng/μL) and PB+urea containing 150 mM urea. Error bars represent SD of three replicates. **(b)** ^{15}N chemical shift differences ($\Delta\delta^{15}$N) obtained from the ^{1}H-^{15}N HSQC spectra. $\Delta\delta^{15}$N= δ^{15}N (MES, pH5.5)- δ^{15}N (MES, pH6.1). **(c)** The NMR signal intensities from ^{1}H-^{15}N HSQC spectra of TDP-43 LCD (100 μm) in pH6.0 10mM phosphate buffer (in red) and TDP-43 LCD (70 μm) in pH5.5 20mM MES buffer (in green) obtained within 2 hours from the sample preparation. **(d)** ^{15}N chemical shift differences ($\Delta\delta^{15}$N) obtained from the ^{1}H-^{15}N HSQC spectra. $\Delta\delta^{15}$N= δ^{15}N (PB)- δ^{15}N (MES, pH5.5). **(e)** The NMR signal intensity changes with time. Red color indicates the residue signal intensity from ^{1}H-^{15}N HSQC spectrum of TDP-43 LCD (100 μM) in pH6.0 10mM phosphate buffer with 150mM urea, obtained within 2 hours from sample preparation (labeled as I_{1h} using the average time) and blue color indicates the spectrum obtained at ~22h from the sample preparation.

Table S1. Amino acid Composition of TDP-43 LCD (a) the full sequence, (b) TDP-43 LCD without the helical segment (residue 320-343), (c) TDP-43 LCD helical segment (residue 320-343)

a. Amino acid Composition of TDP-43 LCD		
**Number of Amino acid Residues**	**Amino acid Type**	**Fractional population**
38	Gly	25.7
23	Ser	15.5
20	Asn	13.5
14	Ala	9.5
12	Gln	8.1
10	Met	6.8
8	Phe	5.4
5	Arg	3.4
4	Leu	2.7
4	Pro	2.7
3	Trp	2
2	Glu	1.4
2	Ile	1.4
1	Asp	0.7
1	Lys	0.7
1	Tyr	0.7
0	Cys	0
0	His	0
0	Thr	0
0	Val	0

b. Amino acid Composition of TDP-43 LCD without helical segment		
**Number of Amino acid Residues**	**Amino acid Type**	**Fractional population**
36	Gly	28.8
20	Asn	16
20	Ser	16
9	Gln	7.2
8	Phe	6.4
7	Ala	5.6
5	Arg	4
5	Met	4
4	Pro	3.2
2	Glu	1.6
2	Ile	1.6
2	Leu	1.6
2	Trp	1.6
1	Asp	0.8
1	Lys	0.8
1	Tyr	0.8
0	Cys	0
0	His	0
0	Thr	0
0	Val	0

c. Amino acid Composition of TDP-43 LCD helical segment		
**Number of Amino acid Residues**	**Amino acid Type**	**Fractional population**
7	Ala	30.4
5	Met	21.7
3	Gln	13
3	Ser	13
2	Gly	8.7
2	Leu	8.7
1	Trp	4.3
0	Arg	0
0	Asn	0
0	Asp	0
0	Cys	0
0	Glu	0
0	His	0
0	Ile	0
0	Lys	0
0	Phe	0
0	Pro	0
0	Thr	0
0	Tyr	0
0	Val	0

Table S2. ^{1}H, ^{15}N chemical shifts from four published work (BMRB 26823, 50154, 26728, 26816).

BMRB: 26823			BMRB: 50154			BMRB: 26728			BMRB: 26816		
20 μM, pH 6.1 20 mM MES, 298K			110 μM, pH4.0, 1 mM CD3COOD, 298K			100 μM, pH 6.5, 10 mM PB, 283K			0.8 mM, pH 2.5, 8 M Urea, 283K		
	^{15}N	^{1}H		^{15}N	^{1}H		^{15}N	^{1}H		^{15}N	^{1}H
-	-	-	266	121.443	8.41	266	118.133	8.619	266	118.135	8.661
-	-	-	267	120.537	8.482	267	120.961	8.578	267	121.438	8.646
-	-	-	268	121.938	8.355	268	121.73	8.418	268	122.091	8.488
269	121.088	8.405	269	120.91	8.369	269	121.193	8.496	269	122.099	8.583
270	122.907	8.2	270	122.796	8.147	270	123.109	8.321	270	124.229	8.446
271	121.834	8.326	271	121.108	8.24	271	121.914	8.455	271	122.005	8.566
272	121.842	8.367	272	121.961	8.334	272	122.152	8.493	272	123.316	8.64
273	116.31	8.278	273	116.406	8.273	273	116.674	8.441	273	117.443	8.57
274	110.444	8.379	274	110.64	8.367	274	110.746	8.517	274	110.828	8.546
275	120.341	8.067	275	120.357	8.071	275	120.582	8.202	275	120.858	8.299
276	120.864	8.315	276	120.915	8.298	276	121.154	8.481	276	121.697	8.545
277	110.717	8.269	277	110.658	8.245	277	111.027	8.403	277	111.178	8.437
278	107.819	7.894	278	107.88	7.891	278	107.975	8.005	278	108.016	7.981
279	119.017	8.336	279	119.052	8.324	279	119.17	8.492	279	119.294	8.583
281	108.617	8.385	281	108.664	8.357	281	108.961	8.537	281	108.78	8.491
282	108.134	8.036	282	108.177	8.029	282	108.33	8.156	282	108.368	8.17
283	119.7	8.138	283	119.704	8.117	283	119.944	8.277	283	119.89	8.309
284	110.247	8.393	284	110.579	8.381	284	110.544	8.536	284	110.482	8.529
285	118.629	8.27	285	118.605	8.259	285	118.788	8.408	285	118.985	8.459
286	120.46	8.452	286	120.495	8.438	286	120.688	8.599	286	120.929	8.629
287	109.388	8.379	287	109.406	8.358	287	109.701	8.515	287	109.646	8.489
288	108.103	8.031	288	108.332	8.098	288	108.457	8.234	288	108.482	8.245
289	119.685	8.141	289	119.748	8.123	289	119.852	8.288	289	119.854	8.313
290	110.247	8.395	290	110.259	8.383	290	110.546	8.538	290	110.517	8.53
291	118.573	8.273	291	118.741	8.257	291	118.898	8.402	291	119.008	8.436
292	116.153	8.31	292	116.216	8.302	292	116.456	8.459	292	116.746	8.519
293	122.429	8.369	293	122.491	8.361	293	122.749	8.522	293	123.019	8.571
294	109.46	8.322	294	109.491	8.308	294	109.762	8.5	294	109.756	8.461
295	108.594	8.26	295	108.652	8.244	295	108.793	8.39	295	108.706	8.375
296	108.51	8.273	296	108.792	8.277	296	108.974	8.424	296	108.915	8.414
297	123.624	8.232	297	123.639	8.214	297	123.756	8.376	297	123.75	8.382
298	107.952	8.355	298	107.967	8.341	298	108.172	8.491	298	108.22	8.484
299	121.267	8.096	299	121.279	8.077	299	121.385	8.24	299	121.46	8.248
300	109.107	8.413	300	109.133	8.388	300	109.368	8.551	300	109.413	8.543
301	118.553	8.272	301	118.472	8.268	301	118.627	8.416	301	118.764	8.458

302	119.004	8.459	302	119.102	8.463	302	119.245	8.599	302	119.632	8.655
303	120.366	8.336	303	120.239	8.351	303	120.585	8.469	303	120.877	8.497
304	109.47	8.387	304	109.473	8.37	304	109.526	8.506	304	109.785	8.487
305	115.406	8.176	305	115.404	8.167	305	115.579	8.314	305	115.509	8.339
306	120.415	8.493	306	120.581	8.481	306	120.673	8.634	306	121.11	8.704
307	120.289	8.303	307	120.314	8.3	307	120.508	8.439	307	120.859	8.489
308	109.539	8.372	308	109.455	8.362	308	109.697	8.461	308	109.782	8.49
309	108.577	8.233	309	108.601	8.219	309	108.758	8.361	309	108.662	8.355
310	108.753	8.29	310	108.596	8.262	310	108.727	8.407	310	108.759	8.407
311	119.353	8.173	311	119.426	8.167	311	119.529	8.315	311	119.722	8.384
312	119.372	8.314	312	119.416	8.313	312	119.621	8.459	312	120.085	8.566
313	120.878	8.214	313	120.861	8.19	313	121.166	8.357	313	121.454	8.416
314	109.861	8.281	314	109.971	8.271	314	110.17	8.413	314	110.15	8.44
315	123.571	7.994	315	123.544	7.972	315	123.73	8.131	315	123.728	8.148
316	118.141	8.025	316	118.211	8.028	316	118.498	8.188	316	119.025	8.269
317	116.568	8.013	317	116.736	8.023	317	117.027	8.145	317	117.694	8.311
318	121.334	7.985	318	121.498	7.999	318	121.858	8.175	318	122.487	8.303
319	123.305	8.245	319	123.345	8.254	319	123.852	8.418	319	123.651	8.614
321	121.188	8.119	321	121.326	8.11	321	121.612	8.243	320	122.774	8.317
322	118.03	7.95	322	118.027	7.942	322	118.56	8.081	322	118.938	8.229
323	120.267	7.96	323	120.355	7.963	323	120.718	8.113	323	121.793	8.345
324	123.387	8.165	324	123.716	8.155	324	123.699	8.309	324	125.596	8.45
325	122.007	8.044	325	122.188	8.064	325	122.353	8.196	325	123.648	8.379
326	121.953	8.044	326	122.102	8.053	326	122.235	8.174	326	123.286	8.357
327	118.046	8.056	327	118.172	8.06	327	118.454	8.173	327	119.514	8.364
328	123.073	8.049	328	123.359	8.06	328	123.326	8.175	328	125.344	8.392
329	121.132	7.914	329	121.333	7.941	329	121.43	8.029	329	123.189	8.315
330	119.77	7.834	330	119.94	7.851	330	120.147	7.948	330	121.683	8.269
331	119.119	8.039	331	119.408	8.063	331	119.407	8.166	331	121.429	8.524
332	115.416	8.104	332	115.662	8.13	332	115.801	8.245	332	117.278	8.485
333	117.15	8.113	333	117.207	8.128	333	117.443	8.233	333	117.904	8.433
334	122.383	8.028	334	122.45	8.027	334	122.664	8.145	334	123.006	8.266
335	109.23	8.167	335	109.443	8.163	335	109.5	8.288	335	110.274	8.325
336	119.571	8.041	336	119.593	8.033	336	119.723	8.161	336	119.828	8.231
337	119.686	8.26	337	119.933	8.257	337	119.947	8.396	337	121.145	8.554
338	109.007	8.257	338	109.141	8.262	338	109.29	8.372	338	109.949	8.472
339	119.635	8.041	339	119.636	8.037	339	119.882	8.166	339	119.99	8.29
340	122.258	8.147	340	122.307	8.144	340	122.621	8.304	340	123.692	8.46
341	124.021	8.191	341	124.21	8.205	341	124.406	8.352	341	125.407	8.505
342	113.746	8.079	342	113.855	8.083	342	114.059	8.225	342	114.989	8.37
343	121.388	8.194	343	121.485	8.192	343	121.634	8.315	343	122.191	8.493

344	120.283	8.228	344	120.366	8.227	344	120.538	8.341	344	121.166	8.471
345	119.228	8.396	345	119.435	8.399	345	119.532	8.541	345	120.225	8.633
346	120.631	8.352	346	120.691	8.342	346	120.915	8.492	346	121.254	8.563
347	116.606	8.337	347	116.635	8.326	347	116.886	8.469	347	117.082	8.498
348	110.451	8.187	348	110.448	8.179	348	110.62	8.318	348	110.457	8.352
350	115.829	8.443	350	115.89	8.42	350	116.206	8.604	349	116.451	8.621
351	110.352	8.322	351	110.361	8.319	351	110.591	8.451	351	110.784	8.571
352	118.509	8.281	352	118.549	8.289	352	118.627	8.41	352	118.689	8.445
353	119.058	8.432	353	119.139	8.424	353	119.315	8.571	353	119.691	8.624
354	120.017	8.362	354	120.122	8.347	354	120.213	8.492	354	120.463	8.512
355	119.027	8.426	355	119.277	8.381	355	119.247	8.557	355	119.625	8.581
356	120.037	8.358	356	120.287	8.321	356	120.317	8.477	356	120.524	8.502
357	109.207	8.408	357	109.247	8.388	357	109.463	8.527	357	109.485	8.505
358	118.515	8.271	358	118.483	8.252	358	118.631	8.388	358	118.767	8.442
359	120.373	8.284	359	120.389	8.281	359	120.613	8.421	359	120.987	8.497
360	121.215	8.329	360	121.126	8.314	360	121.51	8.466	360	121.912	8.547
361	122.319	8.286	361	122.179	8.263	361	122.742	8.443	361	123.112	8.556
362	123.155	8.416	362	122.379	8.352	362	123.5	8.572	362	122.83	8.621
364	117.837	8.47	364	117.945	8.422	364	118.318	8.636	364	119.036	8.642
365	120.584	8.209	365	120.532	8.207	365	120.939	8.366	365	121.17	8.48
366	124.736	8.222	366	124.759	8.199	366	125.054	8.369	366	125.247	8.389
367	119.285	8.141	367	119.359	8.126	367	119.716	8.308	367	119.937	8.34
368	110.616	8.253	368	110.673	8.259	368	110.997	8.392	368	110.909	8.428
369	115.508	8.214	369	115.517	8.209	369	115.707	8.342	369	115.672	8.365
370	110.658	8.46	370	110.671	8.444	370	110.84	8.593	370	110.578	8.47
371	118.496	8.272	371	118.615	8.275	371	118.746	8.392	371	118.778	8.422
372	119.22	8.403	372	119.226	8.429	372	119.442	8.542	372	119.767	8.596
373	115.678	8.19	373	115.734	8.178	373	115.921	8.319	373	116.169	8.366
374	121.963	8.179	374	122.005	8.183	374	122.245	8.319	374	122.493	8.379
375	117.815	8.2	375	117.841	8.194	375	118.199	8.34	375	118.406	8.393
376	110.348	7.863	376	110.368	7.85	376	110.558	7.929	376	110.483	7.881
377	115.372	8.178	377	115.376	8.179	377	115.482	8.313	377	115.346	8.329
378	120.732	8.481	378	120.697	8.49	378	120.998	8.621	378	121.259	8.68
379	116.008	8.263	379	116.033	8.258	379	116.273	8.391	379	116.4	8.424
380	110.697	8.365	380	110.753	8.362	380	110.948	8.485	380	110.909	8.478
381	123.484	7.965	381	123.555	7.954	381	123.629	8.082	381	123.606	8.114
382	123.001	8.128	382	123.063	8.123	382	123.312	8.277	382	123.755	8.334
383	119.454	7.946	383	119.504	7.936	383	119.976	8.125	383	120.454	8.197
384	112.23	8.266	384	112.24	8.243	384	112.638	8.417	384	112.778	8.428
385	121.152	8.025	385	121.176	8.007	385	121.398	8.174	385	121.424	8.224
386	110.661	8.266	386	110.585	8.261	386	110.974	8.384	386	110.885	8.436

387	115.578	8.081	387	115.603	8.072	387	115.805	8.216	387	115.857	8.285
388	125.668	8.353	388	125.702	8.339	388	125.904	8.498	388	126.131	8.529
389	114.408	8.188	389	114.445	8.178	389	114.69	8.323	389	114.896	8.366
390	120.476	8.297	390	120.541	8.291	390	120.75	8.425	390	121.054	8.509
391	124.004	8.188	391	124.029	8.176	391	124.197	8.311	391	124.301	8.329
392	107.838	8.291	392	107.854	8.275	392	108.056	8.414	392	108.055	8.406
393	115.464	8.174	393	115.397	8.156	393	115.644	8.309	393	115.661	8.333
394	110.853	8.459	394	110.818	8.444	394	111.143	8.593	394	111.115	8.587
395	115.516	8.222	395	115.512	8.196	395	115.727	8.362	395	115.674	8.371
396	110.472	8.378	396	110.539	8.366	396	110.659	8.513	396	110.729	8.519
397	120.223	8.11	397	120.117	8.1	397	120.477	8.245	397	120.58	8.294
398	121.471	8.415	398	121.468	8.4	398	121.851	8.564	398	122.18	8.608
399	108.482	7.788	399	108.498	7.765	399	108.622	7.875	399	108.715	7.798
400	108.272	8.106	400	108.182	8.036	400	108.238	8.135	400	108.323	8.189
401	119.905	8.117	401	119.817	8.119	401	120.092	8.256	401	120.15	8.303
402	110.711	8.371	402	110.479	8.374	402	111.02	8.507	402	110.847	8.502
403	115.498	8.173	403	115.517	8.14	403	115.768	8.307	403	115.686	8.306
404	117.557	8.397	404	117.642	8.387	404	117.889	8.549	404	118.213	8.596
405	121.706	8.351	405	121.551	8.303	405	121.922	8.472	405	121.995	8.476
406	120.945	8.2	406	120.178	8.243	406	121.174	8.307	406	119.924	8.499
407	116.564	8.209	407	116.349	8.162	407	116.806	8.329	407	116.807	8.369
408	122.508	8.28	408	122.67	8.257	408	122.776	8.404	408	123.296	8.461
409	116.07	8.173	409	116.158	8.171	409	116.393	8.298	409	116.898	8.433
410	117.426	8.255	410	117.472	8.244	410	117.79	8.41	410	117.913	8.47
411	110.234	8.27	411	110.282	8.255	411	110.505	8.404	411	110.437	8.407
412	120.858	8.002	412	121.007	7.997	412	121.117	8.12	412	121.236	8.187
413	111.395	8.226	413	111.243	8.204	413	111.798	8.356	413	111.038	8.406
414	124.562	7.635	414	123.272	7.705	414	124.642	7.757	414	120.033	8.143

Table S3. ^{1}H, ^{15}N chemical shifts from this research.

TDP-43 LCD	pH5.5 20mM MES (70 μM)		pH6.0 10mM PB (1h) (100 μM)		pH6.0 10 mM PB (22h) (100 μM)		pH6.0 10 mM PB+20ng/mL RNA (100 μM)		pH6.0 10 mM PB+150 mM Urea (100 μM)	
	^{15}N	^{1}H	^{15}N	^{1}H	^{15}N	^{1}H	^{15}N	^{1}H	^{15}N	^{1}H
269	121.19	8.4	121.04	8.41	121.09	8.41	121.04	8.41	121.04	8.41
270	122.85	8.18	122.76	8.17	122.79	8.17	122.76	8.17	122.78	8.17
271	121.77	8.3	121.7	8.3	121.7	8.3	121.7	8.3	121.71	8.31
272	121.82	8.35	121.82	8.35	121.82	8.35	121.82	8.35	121.84	8.36
273	116.46	8.29	116.37	8.28	116.37	8.28	116.37	8.28	116.4	8.29
274	110.48	8.38	110.48	8.38	110.48	8.38	110.48	8.39	110.52	8.39
275	120.39	8.08	120.38	8.08	120.38	8.08	120.38	8.08	120.41	8.08
276	120.86	8.32	120.86	8.32	120.86	8.32	120.86	8.32	120.88	8.33
277	110.72	8.27	110.72	8.27	110.72	8.27	110.72	8.27	110.76	8.27
278	107.87	7.9	107.87	7.9	107.87	7.9	107.87	7.9	107.9	7.91
279	119.05	8.33	119.05	8.33	119.07	8.34	119.07	8.34	119.07	8.35
281	108.67	8.39	108.67	8.39	108.67	8.39	108.68	8.39	108.7	8.39
282	108.18	8.04	108.19	8.04	108.19	8.04	108.19	8.04	108.22	8.05
283	119.7	8.14	119.76	8.13	119.76	8.13	119.77	8.14	119.79	8.14
284	110.28	8.39	110.28	8.39	110.28	8.39	110.32	8.4	110.35	8.4
285	118.63	8.27	118.63	8.27	118.63	8.27	118.63	8.27	118.65	8.28
286	120.46	8.45	120.46	8.45	120.46	8.45	120.48	8.46	120.5	8.46
287	109.44	8.38	109.44	8.38	109.44	8.38	109.46	8.38	109.49	8.38
288	108.15	8.03	108.15	8.03	108.17	8.04	108.15	8.03	108.18	8.04
289	119.69	8.14	119.69	8.14	119.73	8.14	119.7	8.15	119.72	8.15
290	110.29	8.4	110.29	8.4	110.29	8.4	110.3	8.4	110.33	8.41
291	118.57	8.27	118.57	8.27	118.57	8.27	118.57	8.27	118.59	8.28
292	116.2	8.31	116.2	8.31	116.2	8.31	116.22	8.32	116.23	8.32
293	122.43	8.37	122.43	8.37	122.43	8.37	122.48	8.38	122.5	8.38
294	109.5	8.32	109.49	8.33	109.49	8.33	109.49	8.33	109.53	8.33
295	108.63	8.26	108.63	8.26	108.63	8.26	108.66	8.26	108.66	8.26
296	108.55	8.27	108.59	8.27	108.59	8.27	108.6	8.27	108.62	8.27
297	123.62	8.23	123.67	8.23	123.67	8.23	123.67	8.24	123.68	8.24
298	107.99	8.36	107.99	8.36	107.99	8.36	107.99	8.36	108.02	8.36
299	121.27	8.1	121.27	8.1	121.31	8.1	121.31	8.1	121.31	8.1
300	109.16	8.41	109.16	8.41	109.16	8.41	109.16	8.41	109.17	8.42
301	118.55	8.27	118.55	8.27	118.55	8.27	118.55	8.27	118.55	8.28
302	119.04	8.46	119.04	8.46	119.04	8.46	119.07	8.46	119.08	8.47
303	120.38	8.33	120.38	8.33	120.38	8.33	120.38	8.33	120.43	8.34
304	109.51	8.39	109.51	8.39	109.51	8.39	109.51	8.39	109.51	8.39
305	115.45	8.18	115.45	8.18	115.45	8.18	115.45	8.18	115.44	8.18

306	120.42	8.49	120.48	8.49	120.48	8.49	120.46	8.5	120.49	8.5
307	120.33	8.3	120.33	8.3	120.33	8.3	120.33	8.3	120.37	8.31
308	109.54	8.37	109.54	8.37	109.54	8.37	109.54	8.38	109.55	8.38
309	108.63	8.23	108.63	8.23	108.63	8.23	108.62	8.24	108.65	8.24
310	108.8	8.29	108.82	8.29	108.82	8.29	108.82	8.3	108.83	8.3
311	119.35	8.17	119.4	8.17	119.4	8.17	119.38	8.18	119.41	8.18
312	119.42	8.31	119.42	8.31	119.42	8.31	119.42	8.32	119.46	8.32
313	120.9	8.21	120.9	8.21	120.9	8.21	120.89	8.21	120.93	8.22
314	109.89	8.28	109.84	8.28	109.89	8.28	109.91	8.28	109.9	8.29
315	123.61	8	123.61	8	123.61	8	123.61	8	123.63	8
316	118.14	8.03	118.09	8.01	118.14	8.02	118.15	8.02	118.17	8.03
317	116.57	8.01	116.57	8.01	116.61	8.02	116.61	8.01	116.64	8.02
318	121.37	7.99	121.35	7.98	121.35	7.98	121.38	7.99	121.42	8
319	123.34	8.24	123.36	8.22	123.35	8.23	123.39	8.24	123.4	8.25
321	121.24	8.12	121.19	8.12	121.23	8.12	121.22	8.12	121.27	8.12
322	118.09	7.95	118.05	7.95	118.09	7.95	118.1	7.95	118.12	7.96
323	120.3	7.96	120.18	7.95	120.29	7.96	120.3	7.96	120.3	7.96
324	123.34	8.17	123.22	8.16	123.34	8.16	123.33	8.17	123.36	8.17
325	122.01	8.04	121.99	8.03	122.02	8.04	122.03	8.04	121.99	8.03
326	121.95	8.04	121.94	8.05	121.94	8.05	121.97	8.05	121.94	8.05
327	118.05	8.06	117.99	8.07	118.04	8.06	118.06	8.06	118.08	8.07
328	122.99	8.04	122.91	8.04	123.04	8.05	123	8.04	123.06	8.05
329	121.14	7.91	121.14	7.92	121.15	7.91	121.14	7.91	121.19	7.92
330	119.77	7.83	119.77	7.85	119.78	7.84	119.78	7.83	119.84	7.85
331	119.12	8.04	119.01	8.04	119.13	8.04	119.12	8.03	119.15	8.04
332	115.44	8.09	115.38	8.09	115.44	8.1	115.43	8.1	115.46	8.11
333	117.18	8.11	117.18	8.11	117.18	8.11	117.18	8.11	117.21	8.12
334	122.43	8.03	122.42	8.03	122.43	8.03	122.44	8.03	122.42	8.03
335	109.23	8.17	109.17	8.17	109.25	8.17	109.26	8.17	109.26	8.17
336	119.57	8.04	119.59	8.04	119.61	8.04	119.59	8.04	119.61	8.04
337	119.7	8.26	119.61	8.24	119.72	8.25	119.69	8.26	119.73	8.26
338	108.96	8.25	108.88	8.25	108.96	8.25	108.97	8.25	108.98	8.26
339	119.64	8.04	119.67	8.04	119.68	8.04	119.66	8.04	119.67	8.04
340	122.26	8.15	122.17	8.14	122.28	8.14	122.27	8.15	122.3	8.15
341	124.02	8.19	124.01	8.19	124.07	8.19	124.07	8.19	124.09	8.2
342	113.76	8.08	113.76	8.08	113.8	8.08	113.78	8.08	113.81	8.08
343	121.4	8.19	121.4	8.19	121.43	8.19	121.4	8.19	121.44	8.2
344	120.27	8.22	120.27	8.22	120.3	8.23	120.31	8.22	120.33	8.23
345	119.23	8.4	119.23	8.4	119.28	8.4	119.23	8.4	119.27	8.4
346	120.63	8.35	120.63	8.35	120.63	8.35	120.63	8.35	120.69	8.36
347	116.63	8.33	116.63	8.33	116.63	8.33	116.65	8.34	116.67	8.34

348	110.49	8.19	110.49	8.19	110.49	8.19	110.49	8.19	110.51	8.19
350	115.88	8.45	115.88	8.45	115.88	8.45	115.9	8.45	115.91	8.45
351	110.35	8.32	110.35	8.32	110.39	8.32	110.4	8.32	110.41	8.33
352	118.51	8.28	118.51	8.28	118.51	8.28	118.51	8.28	118.55	8.29
353	119.1	8.43	119.1	8.43	119.1	8.43	119.1	8.43	119.12	8.44
354	120.02	8.36	120.02	8.36	120.02	8.36	120.02	8.36	120.04	8.37
355	119.06	8.43	119.06	8.43	119.06	8.43	119.06	8.43	119.09	8.43
356	120.06	8.35	120.06	8.35	120.06	8.35	120.06	8.35	120.08	8.36
357	109.21	8.41	109.21	8.41	109.21	8.41	109.22	8.41	109.23	8.42
358	118.52	8.27	118.52	8.27	118.52	8.27	118.52	8.27	118.52	8.27
359	120.37	8.28	120.37	8.28	120.37	8.28	120.37	8.28	120.41	8.29
360	121.14	8.32	121.22	8.33	121.22	8.33	121.22	8.33	121.27	8.33
361	122.32	8.29	122.32	8.29	122.32	8.29	122.41	8.28	122.42	8.29
362	123.09	8.41	123.14	8.41	123.14	8.41	123.14	8.41	123.21	8.42
364	117.88	8.48	117.86	8.47	117.86	8.47	117.89	8.48	117.93	8.48
365	120.58	8.21	120.58	8.21	120.69	8.21	120.65	8.21	120.68	8.22
366	124.74	8.22	124.73	8.22	124.77	8.22	124.76	8.22	124.8	8.23
367	119.32	8.14	119.28	8.14	119.32	8.14	119.32	8.15	119.35	8.15
368	110.62	8.25	110.62	8.25	110.62	8.25	110.62	8.25	110.64	8.26
369	115.53	8.21	115.53	8.21	115.53	8.21	115.53	8.21	115.56	8.22
370	110.63	8.46	110.63	8.46	110.63	8.46	110.65	8.47	110.65	8.47
371	118.5	8.27	118.5	8.27	118.5	8.27	118.5	8.27	118.53	8.28
372	119.26	8.4	119.26	8.4	119.26	8.4	119.27	8.41	119.3	8.41
373	115.69	8.19	115.69	8.19	115.69	8.19	115.69	8.19	115.67	8.19
374	121.96	8.18	121.96	8.18	121.96	8.18	121.96	8.18	121.99	8.19
375	117.84	8.2	117.84	8.2	117.88	8.2	117.88	8.2	117.89	8.21
376	110.4	7.86	110.38	7.87	110.38	7.87	110.41	7.86	110.42	7.87
377	115.37	8.18	115.37	8.18	115.37	8.18	115.38	8.18	115.38	8.18
378	120.73	8.48	120.78	8.49	120.78	8.49	120.78	8.49	120.8	8.49
379	116.05	8.26	116.05	8.26	116.05	8.26	116.06	8.26	116.07	8.27
380	110.7	8.37	110.7	8.37	110.7	8.37	110.75	8.37	110.73	8.37
381	123.48	7.97	123.48	7.97	123.53	7.97	123.5	7.97	123.53	7.97
382	123	8.13	122.99	8.12	123.01	8.13	123.02	8.13	123.06	8.13
383	119.45	7.94	119.43	7.94	119.47	7.94	119.49	7.95	119.51	7.95
384	112.23	8.27	112.22	8.26	112.22	8.26	112.25	8.27	112.27	8.27
385	121.16	8.03	121.17	8.02	121.17	8.02	121.19	8.03	121.21	8.03
386	110.67	8.26	110.67	8.26	110.67	8.26	110.67	8.26	110.7	8.27
387	115.58	8.08	115.58	8.08	115.58	8.08	115.6	8.09	115.63	8.09
388	125.67	8.35	125.67	8.35	125.71	8.35	125.71	8.36	125.72	8.36
389	114.44	8.19	114.44	8.19	114.44	8.19	114.43	8.19	114.48	8.19
390	120.43	8.29	120.43	8.29	120.43	8.29	120.43	8.29	120.45	8.3

391	124	8.19	123.97	8.18	124.01	8.19	124.01	8.19	124.03	8.19
392	107.85	8.29	107.85	8.29	107.85	8.29	107.89	8.29	107.91	8.29
393	115.5	8.17	115.5	8.17	115.5	8.17	115.52	8.18	115.49	8.18
394	110.9	8.46	110.9	8.46	110.91	8.46	110.92	8.47	110.93	8.47
395	115.55	8.22	115.55	8.22	115.55	8.22	115.55	8.22	115.58	8.23
396	110.53	8.38	110.53	8.38	110.53	8.38	110.54	8.38	110.56	8.38
397	120.22	8.11	120.22	8.11	120.27	8.11	120.22	8.11	120.27	8.12
398	121.42	8.41	121.49	8.41	121.49	8.41	121.51	8.42	121.52	8.42
399	108.48	7.79	108.51	7.79	108.51	7.79	108.52	7.79	108.51	7.79
400	108.31	8.1	108.31	8.11	108.31	8.11	108.34	8.11	108.34	8.11
401	119.87	8.12	119.87	8.12	119.91	8.12	119.87	8.12	119.92	8.13
402	110.71	8.37	110.71	8.37	110.71	8.37	110.71	8.37	110.74	8.38
403	115.56	8.17	115.56	8.17	115.56	8.17	115.57	8.18	115.57	8.18
404	117.6	8.4	117.6	8.4	117.6	8.4	117.61	8.4	117.62	8.4
405	121.75	8.35	121.75	8.35	121.75	8.35	121.75	8.35	121.78	8.36
406	120.95	8.2	120.95	8.2	120.98	8.2	120.96	8.2	120.99	8.21
407	116.56	8.21	116.56	8.21	116.56	8.21	116.6	8.21	116.62	8.21
408	122.51	8.28	122.51	8.28	122.51	8.28	122.51	8.28	122.5	8.29
409	116.05	8.17	116.09	8.17	116.09	8.17	116.12	8.17	116.14	8.18
410	117.46	8.25	117.44	8.25	117.49	8.25	117.47	8.26	117.5	8.26
411	110.31	8.27	110.31	8.27	110.31	8.27	110.33	8.27	110.33	8.27
412	120.88	8	120.88	8	120.93	8	120.91	8	120.93	8.01
413	111.34	8.23	111.39	8.22	111.39	8.22	111.45	8.23	111.45	8.23
414	124.53	7.65	124.57	7.64	124.57	7.64	124.57	7.64	124.6	7.64

Movie S1 (separate file). DIC movie of 100 μM TDP-43 LCD in pH 6.0 10 mM phosphate buffer.

Movie S2 (separate file). Optical tweezer-controlled fusion of two TDP-43 LCD droplets in pH 6.0 10 mM phosphate buffer at 100 μM.

Movie S3a (separate file). DIC movie of 200 μM TDP-43 LCD in pH 6.0 10 mM phosphate buffer.

Movie S3b (separate file). The dilution process from 200 μM of TDP-43 LCD to 100 μM in pH 6.0 10 mM phosphate buffer. An equal volume of blank phosphate buffer was added to 200 μM of TDP-43 LCD solution after 1 minute filming. A continuous change of droplet sizes and density could be observed indicating the blank buffer was slowly diffusing into the solution.

Movie S4 (separate file). DIC movie of 20 μM TDP-43 LCD in pH 6.0 10 mM phosphate buffer.

Movie S5 (separate file). DIC movie of 40 μM TDP-43 LCD in pH 6.0 10 mM phosphate buffer.

Movie S6a (separate file). DIC movie of 100 μM TDP-43 LCD in pH 6.0 10 mM phosphate buffer with yeast RNA (20ng/μL).

Movie S6b (separate file). The process of adding yeast RNA (500ng/μL) to 100 μM TDP-43 LCD in pH 6.0 10 mM phosphate buffer to a final concentration of 20ng/μL was observed by DIC. Yeast RNA was added at 21 second, but a clear change of droplet sizes was observed only after 3 min 33 second.

Movie S7 (separate file). DIC movie of 40 μM TDP-43 LCD in pH 6.0 10 mM phosphate buffer with yeast RNA (20ng/μL).

Movie S8 (separate file). DIC movie of 70 μM TDP-43 LCD in pH 5.5 20 mM MES buffer.

Movie S9 (separate file). DIC movie of 100 μM TDP-43 LCD in pH 6.0 10 mM phosphate buffer with 150mM urea.

Movie S10 (separate file). DIC movie of 100 μM TDP-16E in pH 6.0 10 mM phosphate buffer with yeast RNA (20ng/μL).
